# Coding roles of long non-coding RNAs in breast cancer: Emerging molecular diagnostic biomarkers and potential therapeutic targets with special reference to chemotherapy resistance

**DOI:** 10.3389/fgene.2022.993687

**Published:** 2023-01-06

**Authors:** Dharambir Kashyap, Riya Sharma, Neelam Goel, Harpal S. Buttar, Vivek Kumar Garg, Deeksha Pal, Khairan Rajab, Asadullah Shaikh

**Affiliations:** ^1^ Department of Histopathology, Postgraduate Institute of Medical Education and Research, Chandigarh, India; ^2^ Department of Pulmonary Medicine, Postgraduate Institute of Medical Education and Research, Chandigarh, India; ^3^ Department of Information Technology, University Institute of Engineering & Technology, Panjab University, Chandigarh, India; ^4^ Department of Pathology and Laboratory Medicine, University of Ottawa, Faculty of Medicine, Ottawa, ON, Canada; ^5^ Department of Medical Lab Technology, University Institute of Applied Health Sciences, Chandigarh University, Gharuan, Mohali, India; ^6^ Department of Translational and Regenerative Medicine, Postgraduate Institute of Medical Education and Research, Chandigarh, India; ^7^ College of Computer Science and Information Systems, Najran University, Najran, Saudi Arabia

**Keywords:** long non-coding RNAs, breast cancer, oncogenic lncRNA, tumour supressive lncRNA, non-invasive biomarkers, early diagnosis, chemotherapeutic resistance

## Abstract

Dysregulation of epigenetic mechanisms have been depicted in several pathological consequence such as cancer. Different modes of epigenetic regulation (DNA methylation (hypomethylation or hypermethylation of promotor), histone modifications, abnormal expression of microRNAs (miRNAs), long non-coding RNAs, and small nucleolar RNAs), are discovered. Particularly, lncRNAs are known to exert pivot roles in different types of cancer including breast cancer. LncRNAs with oncogenic and tumour suppressive potential are reported. Differentially expressed lncRNAs contribute a remarkable role in the development of primary and acquired resistance for radiotherapy, endocrine therapy, immunotherapy, and targeted therapy. A wide range of molecular subtype specific lncRNAs have been assessed in breast cancer research. A number of studies have also shown that lncRNAs may be clinically used as non-invasive diagnostic biomarkers for early detection of breast cancer. Such molecular biomarkers have also been found in cancer stem cells of breast tumours. The objectives of the present review are to summarize the important roles of oncogenic and tumour suppressive lncRNAs for the early diagnosis of breast cancer, metastatic potential, and chemotherapy resistance across the molecular subtypes.

## 1 Introduction

Epigenetic dysregulations have a crucial impact on the development and progression of human cancers, including breast cancer (DeVaux and Herschkowitz, 2018; Kumar et al., 2019). Epigenetic modification can change the gene expression without changing the nucleotide sequence of that respective gene (Handy et al., 2011). In addition to gene expression, modifications at the transcriptional and epigenetic levels also work post-transcriptionally and can control the phenotype expression of the protein. Till now, several modes of epigenetic regulation such as DNA methylation (hypomethylation and hypermethylation of gene promotor), histone modifications (methylation or acetylation etc.) (Zaguia et al., 2022), abnormal expression of microRNAs (miRNAs), long non-coding RNAs, and small nucleolar RNAs, have been discovered and documented (Ilm et al., 2016; Shanmugam et al., 2018). Moreover, important roles of each mode have been assessed in diverse pathological conditions (Jaenisch and Bird, 2003; Das and Singal, 2004; Kurdistani, 2007; Goel et al., 2017; Kashyap et al., 2018; Karir et al., 2020; Kashyap and Kaur, 2020).

**FIGURE 1:**
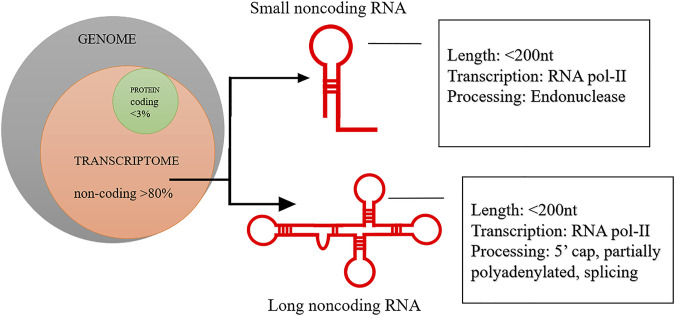
Schematic representation of genomic proportion for coding and noncoding RNA. More than 80% of human genome is noncoding and has genes for different population of noncoding RNA.

Among all epigenetic controls, emerging evidence suggests that lncRNAs can play a crucial role in all types of human cancers, including glioma (Li et al., 2018b), liver (Huang et al., 2020), lung (Chen et al., 2020c), pancreatic (Lv and Huang, 2019), ovarian (Oncul et al., 2020), pancreatic (Pandya et al., 2020), liver (Chen X. et al., 2020) and breast cancer (Koboldt et al., 2012; Kagohara et al., 2018; Kansara et al., 2020; Ghafouri-Fard et al., 2021; Romualdo Cardoso et al., 2022) etc. LncRNAs are one class among different non-coding RNA species uncovered and accounted for ∼80% of the total mammalian genome (Esteller, 2008; Kagohara et al., 2018) (Figure 1). LncRNAs with both oncogenic and tumour suppressive functions have been reported in various human cancers. The lncRNAs have been implicated in regulating the multiple cancer hallmarks, and their associated relationships with apoptosis inhibition, invasion or metastasis initiation, and angiogenesis activation have been demonstrated (Esteller, 2008; DeVaux and Herschkowitz, 2018; Cheng et al., 2019; Kumar et al., 2019; Ma et al., 2019). Notably, the knockdown of oncogenic lncRNA Loc554202 inhibited the proliferation and activated apoptosis of breast cancer cells (Shi et al., 2014). A high expression of lncRNA CBR3-AS1 (AUC ±SD; 0.7 ± 0.05, sensitivity; 0.9, specificity; 0.49, *p* = 0.003) in malignant samples could separate it breast cancer samples from normal control (Hussen et al., 2022). It has been reported that tumour suppressor lncRNAs can inhibit metastasis *via* interacting directly with NF-κB (Liu et al., 2015). Additionally, the presence of lncRNA MEG3 at high levels in breast cancer cells downregulated AKT signalling and modulated the tumour angiogenesis (Zhang C. Y. et al., 2017).

As eluded to earlier, breast cancer is the most common malignancy diagnosed at a high rate in developed and developing countries (Jia et al., 2016; Wang C. et al., 2018; Kashyap et al., 2021a; Tuli et al., 2022). As expected, breast cancer occurs primarily in women (99%) and rarely in men (∼1%–2%). According to GLOBOCAN-2018 report, 2.1 million (11% of total cancer types) new breast cancer cases were diagnosed in 185 countries as opposed to 1.67 million in 2012 (Bray et al., 2018; Guterres and Villanueva, 2020; Kashyap et al., 2021b; 2022a). Despite effective improvement in diagnostic and therapeutic strategies, breast cancer cure remains limited (Nounou et al., 2015). Lack of prognostic and predictive biomarkers information is one reason for failure in early breast cancer detection and management worldwide (Feldman and Kim, 2017; Kashyap et al., 2018; Kashyap and Kaur, 2020). Therefore, specific, accurate, and reliable biomarkers are urgently needed for early detection and effective breast cancer treatment. Based upon the gene expression profiling and immunohistochemistry finding, breast cancer has four molecular subtypes; Luminal A, Luminal B, Her-2 positive and triple negative breast cancer (Kashyap et al., 2022b). The classification of breast cancer based on the expression of estrogen receptor/progesterone receptor, over expression and gene amplification of Her-2 gene (Kashyap et al., 2022b). Patients with each subtype has distinct molecular profile and response to the therapy. Patients with each molecular subtype undergo different targeted therapies (Kashyap et al., 2022b).

A significant amount of data has revealed a link between breast carcinogenesis and dysregulated expression of lncRNAs (Li et al., 2014; Wu et al., 2017). Sophisticated techniques have assessed the aberrant expression of lncRNAs in various breast cancer aspects such as initiation, apoptosis inhibition, metastasis, angiogenesis, and chemotherapy resistance (Gutschner and Diederichs, 2012; Klinge, 2018). LncRNAs also showed differential expression in primary and acquired resistance for radiotherapy, endocrine therapy, immunotherapy, and targeted therapy (Xiu et al., 2019; Du et al., 2020). In addition, many studies have assessed a distinct expression of the vast range of lncRNAs in breast cancer molecular subtypes (Deva Magendhra Rao et al., 2019; DeVaux et al., 2020). A limited number of studies also found lncRNAs with the marked ability for an early breast cancer diagnosis (Jiang et al., 2019; Shin et al., 2019). Moreover, these biomolecules have also suggested to have the capability for changing the expression of cancer stem cell markers in breast tumour (Nie et al., 2018; Bermejo et al., 2019). Previously, studies found polymorphism in long noncoding RNA gene and their association with breast cancer risk (Table 1). The present review will summarize the features and functions of oncogenic and tumour suppressive lncRNAs in early diagnosis of breast cancer, metastatic potential, and underlying mechanism of therapy resistance. In addition, the present will discussion the above-mentioned roles of LncRNA across the different molecular subtypes of breast cancer.

**TABLE 1:** Polymorphism in long noncoding RNA gene and association with breast cancer risk.

Polymorphism	Long non coding RNA	Prognosis	References
rs7158663	LncRNA MEG3	Unfavorable prognostic	Ali et al. (2020)
rs1899663	LncRNA HOTAIR	Unfavorable prognostic	(Lin et al., 2018; Rajagopal et al., 2020)
rs7958904	LncRNA HOTAIR	Unfavorable prognostic	Lin et al. (2018)
rs2839698	LncRNA H19	High risk for breast cancer	Safari et al. (2019) Li et al. (2022)
rs217727	LncRNA H19	High risk for breast cancer	**(** Wang et al., 2019e; Abdelaleem et al., 2021; Li et al., 2022 **)**
rs3741219	LncRNA H19	High risk for ER+ breast cancer	Li et al. (2022)
rs1859168	LncRNA HOTTIP	High risk for breast cancer	Abdelaleem et al. (2021)
rs145204276	LncRNA GAS5	Protective role	(Tang et al., 2019; Sharifi et al., 2020)
rs34841297	LncRNA MIR2052HG	High risk for breast cancer	Yang et al. (2021)
rs920778	LncRNA HOTAIR	High risk for breast cancer	Rajagopal et al. (2020)
rs16949649	LncRNA NME1	High risk for breast cancer	Rajagopal et al. (2020)
rs3827693	LncRNA MALAT1	High risk for breast cancer	Fattahi Dolatabadi et al. (2020)

## 2 Overview of long non-coding RNAs

**FIGURE 2:**
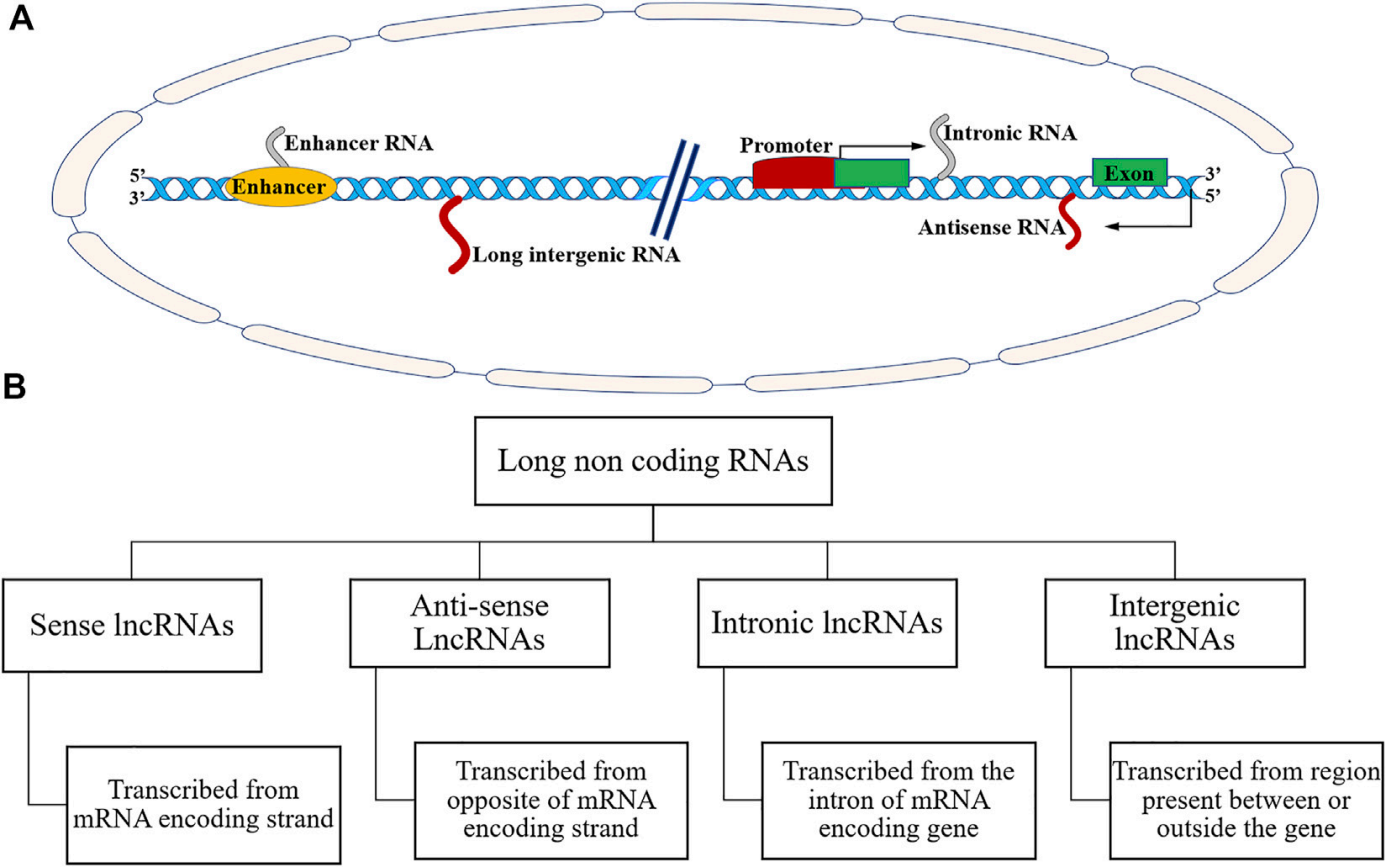
**(A)** Schematic representation of different genomic loci for noncoding RNAs. **(B)** Schematic representation of different types of long noncoding RNAs based on their genomic locus.

**FIGURE 3:**
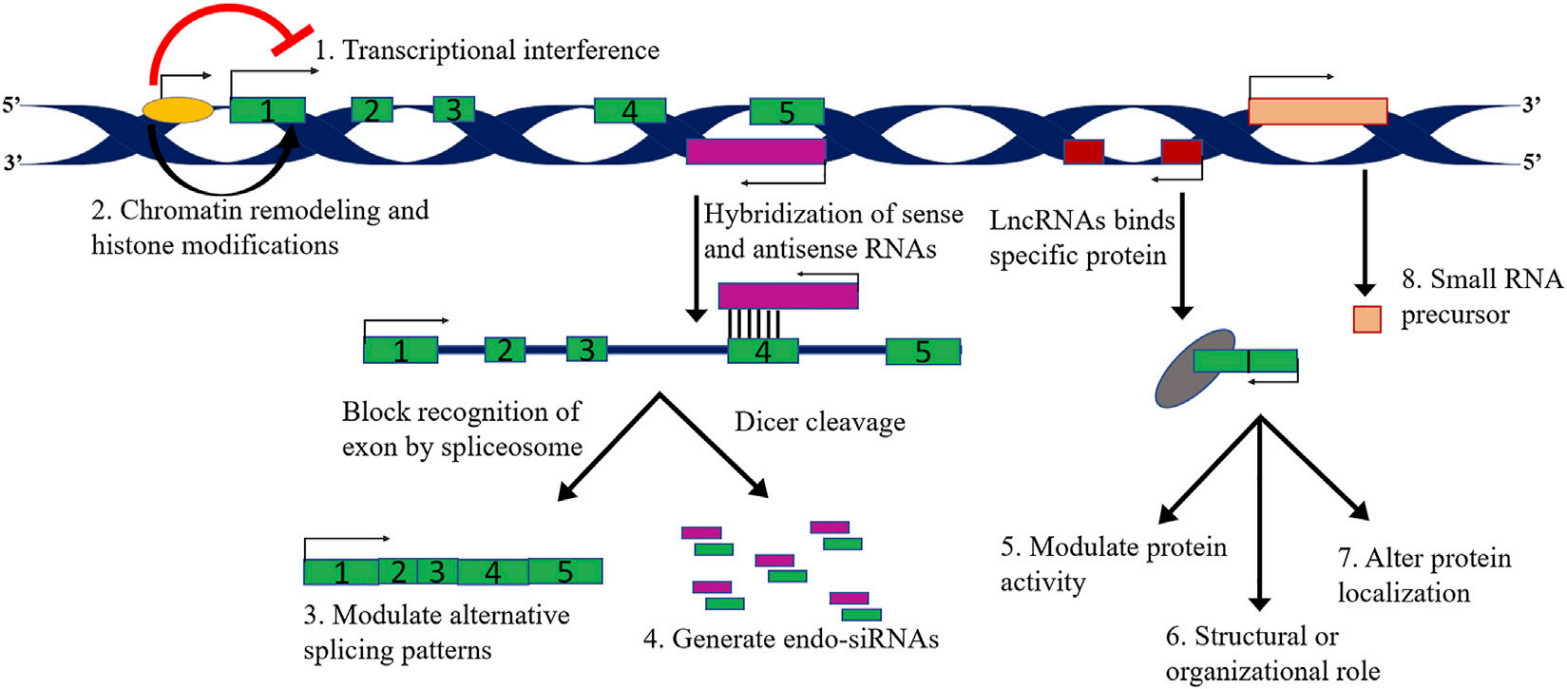
Schematic representation of different types of long noncoding RNAs based on their functions.

Long non-coding RNAs are endogenous non-protein coding RNA biomolecules consisting of 200 bases and 100 kb long (Esteller, 2011). More than 60% of lncRNAs possess a 50-methyl cap at 3′ UTR, and a poly-A tail at 5′ UTR (Cheng et al., 2005; Derrien et al., 2012). The various lncRNAs undergo splicing events and bear one or two exons (Cheng et al., 2005; Derrien et al., 2012). LncRNAs have tissue-specific expression and occupy differential localization in the nucleus and cytoplasm (Fang and Fullwood, 2016; Mishra et al., 2019). Some RNA-seq studies showed that most lncRNAs are poorly conserved in the DNA sequence, whereas other studies noted that several lncRNAs are ultra-conserved in DNA sequence (Necsulea et al., 2014; Fang and Fullwood, 2016). It has been estimated that about 3% of lncRNAs originated more than 300 million years ago and can be found in organisms ranging from Xenopus and chicken to human (Necsulea et al., 2014). Volders et al. have reported as many as ∼60,000 lncRNAs in humans and other mammals (Volders et al., 2019). According to the Encyclopaedia of DNA elements (ENCODE) consortium, there are GENCODE annotated 17,910 lncRNA genes and 48,351 lncRNA transcripts in the human genome (Dunham et al., 2012). Available data indicates that most lncRNAs are transcribed by RNA pol-II (RNA polymerase II) (Kung et al., 2013; Fang and Fullwood, 2016). LncRNAs typically do not possess functional ORFs (open reading frames). The LncRNAs could be transcribed as complex and overlapping transcripts with protein-coding genes (Kung et al., 2013; Fang and Fullwood, 2016). Based on their genomic structure and origin, lncRNAs can be classified into many different types as shown in Figures 2A,B ***.*** These lncRNAs are categorised as: firstly, sense lncRNAs that overlap with one or more exons of another coding gene and transcribed in the same coding gene. Secondly, antisense lncRNA that overlap with one or more exons of a coding gene and are transcribed in the opposite direction of the gene. Thirdly, intronic lncRNAs; located within the introns of protein-coding genes. Fourthly, long intergenic lncRNAs; derived from a genomic sequence between the two coding genes (Kung et al., 2013; Fang and Fullwood, 2016; Huang et al., 2019) (Figures 2, 3). Few pseudogenes, a part of junk DNA, acquire mutations and becomes non-coding sequences, i.e., lncRNAs (Kung et al., 2013). About 20% of human transcriptomes overlap with lncRNA coding sequences (Kung et al., 2013).

Based on the mechanism of action consideration, lncRNAs can be further classified by their interaction with chromatin complexes or directly binding with DNA, organize nuclear architecture by serving as scaffolds, control intracellular trafficking, regulate proteins activities through interplay with cellular macromolecules such as protein complexes and other RNAs (Mohammad et al., 2010; Aguilo et al., 2011; Kotake et al., 2011; Moran et al., 2012; Grote et al., 2013; Kallen et al., 2013; Li et al., 2014; Yang et al., 2014) (see Figure 3; Tables 2, 3; Supplementary Table S1).

**TABLE 2:** Classification of long noncoding RNAs based on their physiological cellular roles.

Type of lncRNAs	Functional role
Guide	Interacts with active enzyme-substrate complexes and directs them to their target site
Dynamic scaffold	Provide a central platform for multiple protein complexes for interactions: including cofactors that direct them to genomic location
Signalling molecule	Part of specific signalling for the activation of molecular pathways
Decoy	Activation and silencing of transcription factors or repressors
miRNA sponge	Host gene for miRNA production
	act as a competitive endogenous RNA (ceRNA) for mRNA degradation by the miRNA complex

**TABLE 3:** Summary of functions, targets, and experimental models used for studying the downregulated long noncoding RNAs in different *in vivo* and *in vitro* investigations.

LncRNA	Status	Target	Consequences	*In vivo*	*In vitro*	Technique	Reference
LncRNA MAGI2-AS3	↓	miR-374	Migration,Invasive	—	MCF-7	qRT-PCR	Du et al. (2019)
					MDA-MB-231		
	↓	FasR,FasL	Migration,Invasive	ER/PR/Her2 (+/-) breast cancer tissues	MDAMB-231	qRT-PCR	Yang et al. (2018b)
					MCF-7		
					MCF-10A		
LncRNA MALAT-1	↓	PI3K-AKT	EMT	ER/PR/Her2 (+/-) breast cancer tissues	MDA-MB-231 MDA-MB-453	qRT-PCR	Xu et al. (2015b)
					BT549		
					SK-BR-3		
	↓	miR-196a-5p	Poor prognosis	TNBC tissues	MDA-MB-231 MDA-MB-468	qRT-PCR	Pickard and Williams, (2014)
					MCF-7		
					T47D		
					BT474		
	↓	—	Inhibit proliferation, & EMT, Increase apoptosis, autophagy, ER stress, regulate Akt/mTOR pathway, & p38 MAPK/Erk signaling	—	MCF-7	qRT-PCR	Huang Y et al. (2018)
LncRNA EGOT	↓	—	Poor prognosis	ER/PR/Her2 (+/-) breast cancer tissues	—	qRT-PCR	Xu et al. (2015a)
LncRNA LINC00628	↓	—	Poor prognosis	Breast cancer tissue	MDA-MB-231	qRT-PCR	Chen et al. (2017a)
					HCC1937		
					LCC9		
					LCC2		
					MCF-7		
LncRNA FGF14-AS2	↓	miR-205-5p	Poor prognosis	Breast cancer tissues	MDA-MB-231	qRT-PCR	Yang et al. (2019b)
					SK-BR-3		
LncRNA MEG3	↓	—	Increase proliferation, angiogenesis through regulating AKT signaling	BALB/c nude mice	MDA-MB-231	qRT-PCR	Zhang et al. (2017a)
					MCF-7		
LncRNA TUSC8	↓	miR-190b-5p	Increase Metastasis, & EMT	TNBC tissues	MDA-MB-231	qRT-PCR	Zhao et al. (2020)
					MCF-7		
					SK-BR-3		
LncRNA CTD-2108O9.1	↓	LIFR	Suppress metastasis	ER/PR/Her2 (+/−) breast cancer tissues & female balb/c nude	MCF-7	qRT-PCR	Wang et al. (2018c)
					MDA-MB-231		
LncRNA LINC01121	↓	—	Inhibit proliferation, & EMT, Increase apoptosis, autophagy, ER stress, regulate Akt/mTOR pathway, & p38 MAPK/Erk signaling	—	MCF-7	qRT-PCR	Huang Y et al. (2018)
LncRNA PTTG3P	↓	—	Inhibit proliferation, & EMT, Increase apoptosis, autophagy, ER stress, regulate Akt/mTOR pathway, & p38 MAPK/Erk signaling	—	MCF-7	qRT-PCR	Huang Y et al. (2018)
LncRNA CASC2	↓	miR-96-5p	Regulate expression of SYVN1 gene, & Decrease apoptosis	Breast cancer tissue	MDA-MB-231	qRT-PCR	Gao et al. (2018)
					MCF-7		
	↓	miR-18a-5p	Paclitaxel resistance through regulating CASC2/miR-18a-5p/CDK19 axis	Breast cancer tissue & male	MDA-MB-231	qRT-PCR	Zheng et al. (2019c)
					MCF-7		
				BALB/c nude mice			
ncRNA lncFOXO1	↓	—	Bind with BRCA-1, and regulate	Breast cancer tissues	MDA-MB-231 MDA-MB-453 MDA-MB-415	qRT-PCR	Xi et al. (2017)
					MCF-7		
					BT-549		
			H2A				
LncRNA 00641	↓	miR-194-5p	Increase proliferation, migration, invasion, inhibit apoptosis	Breast cancer tissues	MDA-MB-453 UACC-812	qRT-PCR	Mao et al. (2020)
					MDA-MB-231		
					BCAP-37		
					MCF-7		
LncRNA TFAP2A-AS1	↓	miR-933	Regulate cell cycle, apoptosis by miR-933/SMAD2 axis	Breast cancer tissues	MDA-MB-231 MDA-MB-435 MCF-10A	qRT-PCR	Zhou et al. (2019a)
					MCF-7		
					T-47D		
					SKBR-3		
LncRNA FGF14-AS2	↓	miR-370-3p	Cancer growth by regulating FGF14-AS2/miR-370-3p/FGF14 axis	Breast cancer tissues	MDA-MB-453	qRT-PCR	Jin et al. (2020)
					MDA-MB-231		
					MCF-7		
					HCC-1937		
LncRNA AC073284.4	↓	miR-18b-5p	Paclitaxel resistance, and EMT by regulating DOCK4 gene expression	Breast cancer tissues	MCF-7	qRT-PCR	Wang et al. (2019h)
					SKBR-3		
LncRNA LINC00968	↓	—	Regulate Wnt2/β-catenin signaling pathway	Breast cancer tissues & Breast cancer sample data from GSE26910 & BALB/c nude mice	—	qRT-PCR	Xiu et al. (2019)

Note: ER/PR (+/−) breast cancer (luminal A), ER/PR/Her2 (+/−) (Her2 positive), TNBC (triple negative breast cancer), qRT-PCR (quantitative Real-Time PCR), ISH (*In situ* hybridization), FISH (Fluorescent *in situ* hybridization), SSH (Suppression subtractive hybridization), TGF-β (Transforming growth factor beta), NF-κβ (Nuclear factor-κ β), IKK (Iκ β kinase), EZH2 (Enhancer of zeste homolog 2), CDK6 (Cyclin-dependent kinase 6), Hsp90 (heat shock protein 90), PI3K (phosphatidylinositol 3 kinase)/AKT (protein kinase B), TEAD (Transcriptional enhanced associate domain), PABPC1 (Polyadenylate-binding protein cytoplasmic 1), SRY (sex determining region Y)-box 2, IGF2 (Insulin-like growth factor 2), BTG3 (BTG Anti-Proliferation Factor 3), NONO (Non-POU Domain Containing Octamer Binding), QKI (QKI, KH Domain Containing RNA Binding, RBMX (RNA Binding Motif Protein X-Linked), KLHDC7B (Kelch Domain Containing 7B), HMMR (Hyaluronan Mediated Motility Receptor), LIFR (LIF Receptor Subunit Alpha), SNCG (Synuclein Gamma), POSTN (Periostin), FAT4 (FAT Atypical Cadherin 4), USP7 (Ubiquitin carboxyl-terminal hydrolase 7), HIF-1α (hypoxia-inducible factor 1 alpha), BLCAP (BLCAP Apoptosis Inducing Factor), ABCB1 (ATP Binding Cassette Subfamily B Member 1), Nrf2 (nuclear factor erythroid 2–related factor 2), EMT (epithelial-mesenchymal transition) ER (endoplasmic reticulum stress), MAPK (mitogen-activated protein kinase), MMP 9 (Matrix metallopeptidase-9), IL6)Interleukin-6), ERBB2 (Erb-B2 Receptor Tyrosine Kinase 2), CHST15 (carbohydrate sulfotransferase 15), Oct-4 (octamer-binding transcription factor 4), RUNX2 (Runt-related transcription factor 2), HOXB8 (Homeobox B8), SOX4 (SRY-Box Transcription Factor 4), DNMT1 (DNA Methyltransferase 1), STAT3 (Signal transducer and activator of transcription 3), SAHH (S-adenosylhomocysteinehydrolase), DNMT3B (DNA Methyltransferase 3 Beta), KLF4 (Kruppel Like Factor 4), ALDH1A1 (Aldehyde Dehydrogenase 1 Family Member A1), FGF7 (Fibroblast Growth Factor 7), SYVN1 (synoviolin 1), CASC2 (Cancer Susceptibility 2), EREG (Epiregulin), CCND! (Cyclin D1 gene), BRCA1 (breast cancer 1), IGF2BP1 (Insulin-Like Growth Factor 2 MRNA Binding Protein 1), LEF1 (Lymphoid Enhancer Binding Factor 1), CHEK2 (Checkpoint kinase 2), PTEN (Phosphatase and tensin homolog), ADAM10 (ADAM metallopeptidase domain 10), PKM2 (Tumour M2-pyruvate kinase 2), NOD2 (Nucleotide-binding oligomerization domain-containing protein 2), IGF1R (Insulin-Like Growth Factor 1 Receptor), KPNA2 (Karyopherin Subunit Alpha 2), SNHG22 (Small Nucleolar RNA Host Gene 22), CCR2 (C-C Motif Chemokine Receptor 2), ZEB1 (Zinc Finger E-Box Binding Homeobox 1), FAT4 (FAT Atypical Cadherin 4), SIRT1 (Sirtuin 1), BAALC (BAALC Binder Of MAP3K1 And KLF4), Focal adhesion kinase (FAK), Krüppel-like factor 4 (KLF4), DOCK (dedicator of cytokinesis), SCN3A (Sodium channel, voltage-gated, type III, alpha subunit), ITGB1 (Integrin Subunit Beta 1), OTX1 (Orthodenticle Homeobox 1).

## 3 Functions of lncRNAs

**FIGURE 4:**
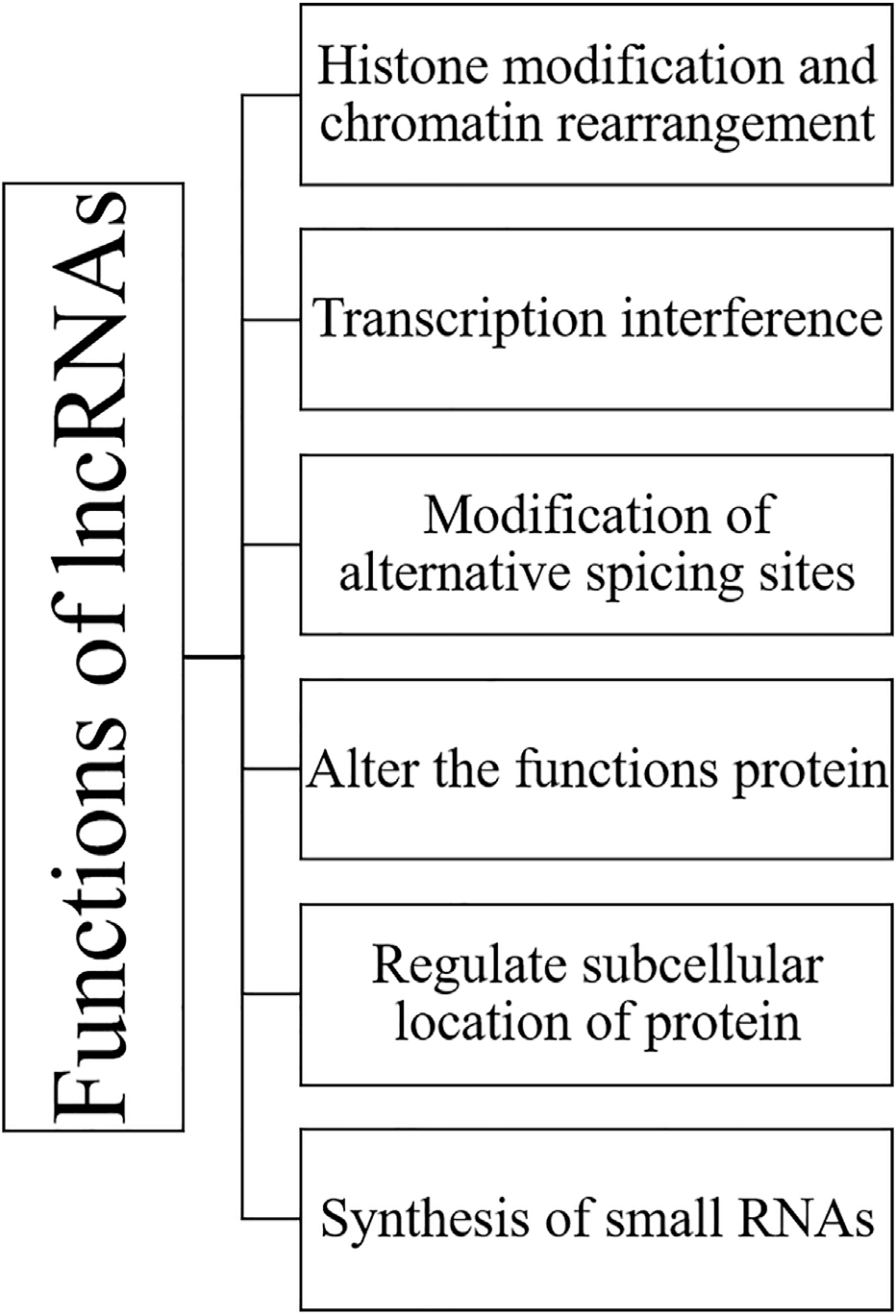
Showing different functions of long noncoding RNAs.

**FIGURE 5:**
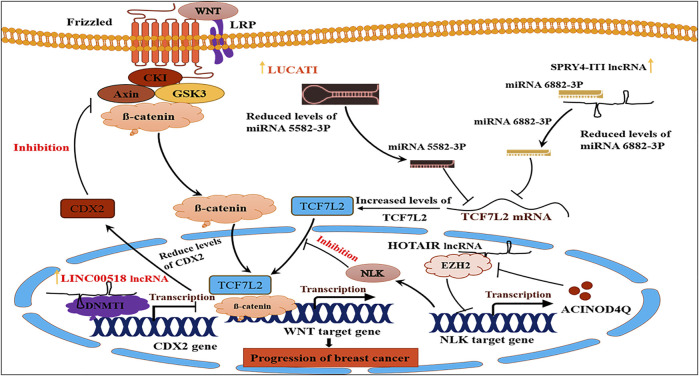
Showing long noncoding RNAs mediated different cancer related signaling pathways. Adapted from (Ghafouri-Fard et al., 2021).

LncRNAs mainly have tissue-specific expression and are expressed at a low level compared to protein-coding genes. LncRNAs work in a complicated way as a critical regulator of epigenetic modulation, transcription, and translation in a spatiotemporal manner. RNA is dynamic transcripts and can form several secondary structures, thus leading to their binding and interactions with a vast range of substrates. LncRNAs can regulate gene expression interfering at pre-and post-transcriptional levels. Based on the mode of regulation, lncRNAs are divided into cis-acting and trans-acting categories; cis-acting lncRNAs: regulate those genes which are present on the same chromosome of their origin; however, trans-acting lncRNAs: regulate a broader range of genes on neighbouring or distant chromosomes. Various functions of lncRNAs are summarized in Figure 4 and Figure 5.

## 4 Long non-coding RNAs in breast cancer pathogenesis

### 4.1 Oncogenic long non-coding RNAs

Oncogenic lncRNAs express at a high-level during carcinogenesis compared to normal conditions and execute oncogenic functions either by interacting with miRNAs or protein molecules. Numerous oncologist researchers and clinicians evaluated the expression of oncogenic lncRNAs and correlated with adverse clinical features of breast cancer patients. For instance, an elevated expression of lncRNA UCA1 under the influence of macrophages infiltration was measured and correlated with the advanced breast cancer clinical stage (Chen et al., 2015). Oncogenic lncRNA PRNCR1 had higher expression in the advanced clinical stage and metastasis positive breast cancer tissues. The silencing of PRNCR1 in an *in-vitro* model reversed its oncogenic effect (Guo et al., 2019). For instance, expression of lncRNA MALAT1 was found to be significantly up-regulated in clinical breast cancer samples and negatively correlated with overall survival in *in-situ* carcinoma. Furthermore, bioinformatics prediction indicated that MALAT1 could regulate BLCAP mRNA through binding with miR-339-5p (Zheng L. et al., 2019). Another study assessed lncRNA CCAT1 overexpression in lymph node metastasis breast cancer tissues. Kaplan-Meier and multivariable analysis correlated lncRNA CCAT1 expression with decreased OS (overall survival) and progression-free survival (PFS) (Zhang et al., 2015). Similarly, higher expression of lncRNA Z38 was associated with large tumour size and lymph node metastasis. Cox regression model predicted lncRNA Z38 as an independent prognostic factor for OS (HR = 4.74, 95% CI 2.41–9.32) with 78% sensitivity and 70% specificity in ROC curve analysis (Li et al., 2018a; Nie et al., 2018), found upregulation of lncRNA LINC00310 and c-Myc in TCGA (The Cancer Genome Atlas) data (Li et al., 2018a). Another study found a remarkably higher expression of lncRNA SNHG7 in breast cancer tissues compared to adjacent normal part. The miRNA-381 considered a tumour suppressor, was found a direct target of lncSNHG7 (Gao and Zhou, 2019). Two lncRNAs that are up-regulated in breast cancer are LUCAT1 (Zheng A. et al., 2019) and SPRY4-IT1 (Song et al., 2020). These lncRNAs, respectively, block the expression of miR-5582-3p and miR-6882-3p. TCF7L2 is upregulated when these miRNAs are downregulated. This factor enhances the expression of genes involved in the development of breast cancer in conjunction with β-catenin. Fan et al. (2017) measured the expression of lncRNA TUG1 in cancer tissue and observed its upregulation and association with poor clinical features (large tumour size, distant metastasis, and TNM (tumour (T), nodes (N), and metastases (M)) (Fan et al., 2017). LncRNA LINP1 also appeared to have higher expression in breast cancer tissues than in adjacent non-tumour tissues (*p* < 0.01) and correlated with advanced TNM stage (*p* = 0.002), poorer pathological differentiation (*p* = 0.004), and shorter overall survival (SOS) and disease-free survival (DFS) (Liu et al., 2018). A meta-analysis determined a negative relation between lncRNA MALAT1 expression and bad prognosis or adverse clinicopathological features. The report demonstrated that elevation in MALAT1 expression significantly predicted unfavourable OS (HR = 2.06, 95% CI: 1.66–2.56, *p* < 0.0001) in progesterone receptor (PR) (OR = 1.47, 95% CI: 1.18–1.82) positive cancer tissues (Wang Y. et al., 2020). Moreover, in another meta-analysis, overexpression of lncRNA HOTTIP predicted the worst clinical outcome (95% CI 1.72–3.03, *p* < 0.00001). Moreover, validation using gene expression omnibus data sets (GSE20711, GSE16446, and GSE9195) and 100 breast cancer patients confirmed similar results (Yang et al., 2017). Based on the expression pattern of four lncRNAs U79277, AK024118, BC040204, and AK000974, Meng et al. (2014) stratified the breast cancer patients into the high-risk and low-risk group (Meng et al., 2014). Higher expression of lncRNA LINC00473 suppressed miR-497 in breast cancer samples and cell lines compared to breast epithelial cells. Multivariate logistic regression assays further suggested LINC00473 as an independent prognostic factor (Bai et al., 2019). Additionally, Cox regression analysis confirmed LINC01296 as an independent prognostic in breast cancer (Jiang M. et al., 2018). Chen et al. (2020b) validated expression of seven lncRNAs (ST8SIA6-AS1, lnc-HIST1H2BJ-5:1, lnc-PRICKLE2-3:2, RP1-86C11.7, RP11-15F12.1, ZNF670-ZNF695, and lnc-STRN3-12:1) in breast cancer. Only the higher expression of ST8SIA6-AS1 was associated with TNM staging and Ki67 index. Hypothetically, lncRNA ST8SIA6-AS1 binds with miR-4252 or interacts with NONO (Non-POU Domain Containing Octamer Binding), QKI (QKI, KH Domain Containing RNA Binding), and RBMX (RNA binding motif protein X-linked) (Chen et al., 2020b). Further, univariate and multivariate COX regression analyses proposed oncogenic lncRNA BANCR as an independent risk factor of poor prognosis (Jiang J. et al., 2018).

In addition, oncogenic lncRNAs can also regulate EMT (Epithelial to mesenchymal transition) during cancer progression. LncRNA HOXD-AS1 interacts with miR-421 and inhibits its expression leading to the upregulation of SOX4, a master regulator of EMT (Li et al., 2019d). Similarly, upregulated lncRNA linc00617 can also promote breast cancer cell motility and EMT process by modulating the Sox2 [(sex-determining region Y)-box 2] gene expression (Li et al., 2017a). The study by He and Wang (2015) found higher expression levels of lncRNA-AK058003 that promoted breast cancer cell proliferation and EMT *via* the regulation of SNCG (Synuclein Gamma) expression (He and Wang, 2015). High expression of lncRNA NEAT1 predicted poor overall survival in breast cancer patients, and silencing of lncRNA NEAT1 suppressed the EMT process through upregulation of miR-146b-5p (Li et al., 2020a). Zheng et al. (2019d) showed that lncRNA RHPN1-AS1 silencing resulted in decreased expression of EMT markers (Zheng S. et al., 2019). In addition, treatment with Pterostilbene increased the expression of the lncRNAs MEG3, TUG1, H19, and DICER1-AS1, whereas decreased the expression of lncRNA LINC01121, PTTG3P, and HOTAIR. Differential expression of these lncRNAs caused inhibition of cell proliferation and EMT (Huang Y et al., 2018). Also, higher expression of lncRNA LINC00673 influenced NCR3LG1 (natural killer cell cytotoxicity receptor 3 ligand 1) activity and enhanced EMT process in breast cancer (Hou et al., 2018). Si et al. (2019) determined upregulation of lncRNA H19 in breast cancer patients with poor prognosis and silencing of lncRNA H19 inhibited the tumour growth EMT (Si et al., 2019). Further, overexpressed lncRNA FOXD2-AS1 regulated the expression of EMT markers (N-cadherin, E-cadherin, and vimentin) *via* the FOXD2-AS1/miR-150-5p axis (Jiang et al., 2019).

Further, oncogenic lncRNAs can also regulate the cell proliferation by controlling the cell division (Rakhshan et al., 2022). For instance, the study by Wu et al. (2017) evaluated the high levels of lncRNA CCAT2 in breast cancer and downregulation of lncRNA CCAT2 arrested the cells in the G0/G1 phase and promoted apoptosis by modulating the TGF-β signalling pathway (Wu et al., 2017). Huang et al. (2014) demonstrated that hnRNP I formed a functional ribonucleoprotein complex with lncRNA UCA1 and leading to an increase in the UCA1 stability. Without lncRNA UCA1, hnRNP I enhanced the translation of p27 and supported the cancer proliferation (Huang et al., 2014). Microarray experiment identified upregulation of lncRNA NONHSAT028712 in breast cancer. LncRNA NONHSAT028712 bound with heat-shock protein 90 (HSP90) and recruit cycle 37 (Cdc37). Mechanistically, lncRNA NONHSAT028712/HSP90/Cdc37 complex activated the cyclin-dependent kinase 2 (CDK2) and regulated the cell cycle (Cui et al., 2020). Depletion of oncogenic lncRNA PRNCR1-2 in HS-578T and MDA-MB-231 breast cancer cells markedly suppressed proliferation rates and cell cycle progression *via* increasing Checkpoint kinase 2 (CHK2) phosphorylation (Pang et al., 2019).

In addition, lncRNAs also regulate the apoptosis activation in breast cancer cells. Breast cancer patients with low expression of lncRNA BANCR were significantly different than patients with higher expression of lncRNA BANCR. Western blotting revealed that of Bax (B-cell lymphoma 2 associated X protein), PARP (cleaved-Caspase-3 and cleaved-poly adenosine diphosphate-ribose polymerase) had elevated expression in low expression lncRNA BANCR group (Jiang J. et al., 2018). Liu et al. (2020) reported that blocking the expression of lncRNA TP73-AS1 in breast cancer cells promoted apoptosis, and inhibited proliferation *via* lncRNA TP73-AS1/miR-125a/MTDH pathway (Liu et al., 2020). Elevated levels of lncRNA HOTAIR were positively associated with Bcl-w positivity in clinical breast cancer samples. The results of another study showed that HOTAIR bound with miR-206 leading to the expression of Bcl-w (Ding et al., 2017). Further, Deng et al. (2016) demonstrated that inhibition of lncRNA Z38 expression by siRNAs treatment suppressed the breast cancer cell tumourigenesis and induced cell apoptosis (Deng et al., 2016). The expression of lncRNAs Loc554202 was significantly increased in breast cancer tissues compared to normal controls. On the other hand, knockdown of Loc554202 had a reversed effect and resulted in inhibition of proliferation and apoptosis activation (Shi et al., 2014).

It was observed that lncRNAs can also regulate several cancers associated signalling pathways, including the activation of transcription factors, such as nuclear factor kappa B (NF-κB). For example, overexpressed lncRNA NKILA bound to NF-κB/IĸB masked its phosphorylation. This interaction prevented the over-activation of the NF-κB pathway in inflammation stimulated breast epithelial cells (Liu et al., 2015). According to Dong et al. (2020) overexpressed lncRNA LOXL1-AS1 sponged miR-708-5p and increased the levels of NF-κB, leading to increased migration and invasion of breast cancer cells (Dong et al., 2020). It has been documented that estrogen receptor-α upregulates lncRNA LINC00472, which subsequently suppresses the phosphorylation of NF-κB (Wang Z. et al., 2019). Similarly, Cao et al. (2019) observed an enhanced expression of lncRNA UASR1 and pAkt, pTSC2, p4EBP1, and p70S6K in breast cancer cells, thereby suggesting that UASR1 played an oncogenic role in breast cancer cells through activation of the Akt/mTOR signalling pathway (Cao et al., 2019).

Bai et al. (2018) found that lncRNA EZR-AS1 interacts with β-catenin to prevent its degradation and lncRNA EZR-AS1 knockout resulted in β-catenin downregulation and inactivation of the Wnt/β-catenin pathway (Bai et al., 2018). Zhao et al. (2019a) reported that lncRNA HEIH regulates miR-200b and may contribute to breast cancer *via* modulation of miR-200b/axis/Wnt/β-catenin pathway (Zhao et al., 2019a). LncRNA RPPH1 overexpression promoted cell cycle and proliferation and increased colony formation by downregulating miR-122. The downregulation of miR-122 results in increase ADAM10 (ADAM metallopeptidase domain 10), PKM2 (Pyruvate kinase M2), NOD2 (Nucleotide-binding oligomerization domain-containing protein 2), and IGF1R (Insulin-like growth factor 1 receptor) genes expression (Zhang and Tang, 2017). Zheng et al. (2019) also observed higher expression lncRNA LUCAT1 in breast cancer cases, whereby downstream inhibited target TCF7L2 (transcription Factor-7-Like 2) gene and activated Wnt/β-catenin pathway (Zheng A. et al., 2019). Upregulation of lncRNA CRNDE inhibited miR-136, leading to upregulation of β-catenin and the activation of the Wnt/β-catenin signalling pathway (Zheng A. et al., 2019).

In 2018, Hou et al. suggested that overexpression of lncRNA ROR promoted proliferation and invasion of cancer cells in nude mice breast model through TGF-β (Transforming growth factor beta) signalling pathway (Hou et al., 2018). Dysregulated lncRNA HOXA-AS2 endogenously sponged miR-520c-3p and caused downregulation of miR-520c-3p that influenced the expression of TGF-β-R2 in breast cancer cells (Fang et al., 2017). Additionally, lncRNA-NORAD promoted proliferation by activation of the TGF-β/RUNX2 signalling pathway in breast cancer cells (Zhou K. et al., 2019). Similarly, highly expressed lncRNA DLX6-AS1 targeted miR-505-3p and subsequently enhanced the expression of the RUNX2 (Runt-related transcription factor 2) gene (Zhao et al., 2019b). In another study, the overexpressed LINC01614 group activated networks of TGF-β1 and ECM (Extracellular matrix) in HR+/HER2+ breast cancer molecular subtype (Vishnubalaji et al., 2019).

Niu et al. (2019) demonstrated that lncRNA LINC00473 could sequester miR-198 and regulate the MAPK1 (Mitogen-activated protein kinase 1) gene expression (Niu et al., 2019). Overexpressed lncRNA SNHG6 inhibits miR-26a-5p and leads to upregulation of MAPK6 (Lv et al., 2019). Furthermore, lncRNA linc01561 caused upregulation of MMP-11 (Metalloproteinase-11) after targeting miR-145-5p in breast cancer cells (Jiang R. et al., 2018). It was found that LncRNAs can also control cancer cell metabolism, *viz.*, lncRNA YIYA regulates CDK6 (cell division protein kinase 6) dependent phosphorylation of PFKFB3 (fructose bis-phosphatase PFK2), and thus can convert glucose 6-phosphate (G6P) to fructose-2,6-phosphate (Jiang R. et al., 2018).

A study by Qian et al. (2017) revealed that lncRNA NEAT1 could promote cancer cell growth through the upregulation of EZH2 (Enhancer of zeste homolog 2) gene by targeting miR-101 (Qian et al., 2017). Higher expression of lncRNA DANCR in advanced tumour grades or lymph node metastasis cases promoted the binding of EZH2 to the promoter region of SOCS3 (Suppressor of cytokine-3 signalling) and inhibited SOCS3 expression (Zhang K. J. et al., 2020). A study by Zhu et al. (2019b) demonstrated that lncRNA linc00460 target miR-489-5p and hence regulate the expression of FGF7 (Fibroblast growth factor 7) and Akt (protein kinase B) (Zhu et al., 2019b). Oncogenic lncRNA FGF14-AS2 suppressed miR-370-3p expression and consequently led to the activation of FGF14 in breast cancer cells (Jin et al., 2020).

Immunoprecipitation assays provided evidence that lncRNA H19 regulated the expression of *STAT3 (Signal transducer and activator of transcription 3)* gene in breast cancer (Li et al., 2019a). The results of Liang et al. (2018b) revealed that lncRNA-PRLB could regulate the chemoresistance in breast cancer *via* modulating the expression of miR-4766-5p and SIRT1 (Sirtuin 1) genes (Liang et al., 2018b) Higher expression levels of RHPN1-AS1 were measured by RNA FISH (fluorescent *in situ* hybridization) and Western blot assays in MCF-7 and MDA-MB-231 breast cancer cell. Luciferase reporter assay validated that RHPN1-AS1 inhibits miR-4261 and regulates the direct transcriptional target of c-Myc (Zhu et al., 2019a).

A number of studies have shown high levels of lncRNA UCA1 in breast cancer tissues, which resulted in tumourigenesis through inhibition of tumour suppressor miRNA-143 (Chen et al., 2015; Tuo et al., 2015). Additional findings from xenograft breast cancer model identified upregulation of lncRNA HOTAIR and chondroitin sulfotransferase CHST15 (GalNAc4S-6ST) (Liu et al., 2019). Further, RNA FISH revealed amplification of lncRNA ANRIL in malignant breast cells. LncRNA amplification was positively correlated with POSTN (periostin) expression (*p* = 0.0086) (Mehta-Mujoo et al., 2019). Huang and Xue suggested the upregulation of lncRNA FOXD2-AS1 in breast cancer cell lines and its positive relationship with S100A1 (Calcium-binding protein A1) gene expression. It was reported that lncRNA FOXD2-AS1/S100A1/Hippo axis was involved in tumourigenesis of breast cancer (Huang and Xue, 2020). Another study also found the elevated expression of lncRNA GHSROS in the cancer cells and its association with the cell migration in *in-vivo* and *invitro* systems (Thomas et al., 2019). According to Li and co-workers, lncRNA H19 also promoted breast cancer growth through H19/miR-152/DNMT1 axis (Li Z. et al., 2017).

Oncogenic lncRNA LINC02163 was found to be involved in breast cancer pathogenesis by mode of LINC02163/miR-511-3p/HMGA2 (high mobility group A proteins 2) axis (Qin et al., 2020). The findings of another study showed that lncRNA LINC00461 regulated KPN-α2 (Karyopherin alpha 2) gene expression through sponging miR-144-3p in the breast cancer (Zhang Q. et al., 2020). Upregulated lncRNA BLACAT1 was linked with aggressive breast cancer phenotype by lncRNA BLACAT1/miR-150-5p/CCR2 (C-C chemokine receptor type 2) axis (Hu et al., 2019).

Li and et al. found that upregulation of lncRNA ZFHX4-AS1 suppresses FAT4 and increases YAP1 (yes-associated protein 1) and TAZ (Tafazzin) gene expression which is attributed to breast cancer cell proliferation (Li et al., 2019b). A report by Wu et al. indicated that high expression of lncRNA HOXA-AS2 might modulate the expression of SCN3α (Sodium voltage gated channel alpha subunit 3) after sponging miR-106a in breast cancer (Li et al., 2019b). Greater expression of lncRNA ADPGKAS1 predicted poor prognosis for breast cancer patients mechanistically by modulating miR-3196/OTX1 axis (Yang J. et al., 2019). Wang et al. (2019c) suggested that lncRNA HULC in breast cancer tissues and cell lines paired with miR-6754-5p and upregulated LYPD1 (LY6/PLAUR domain containing 1) gene expression (Wang et al., 2019c). Vennin et al. (2017) demonstrated that oncogenic lncRNA 91H prevents histone and DNA methylation on the maternal allele at the H19/IGF2 (Insulin Like Growth Factor 2) locus (Vennin et al., 2017).

### 4.2 Tumour suppressive long non-coding RNAs

There are a number of lncRNA whose downregulation contributes in breast cancer development and progression (Ghafouri-Fard et al., 2022). A meta-analysis by Xu et al. (2016) on two cohorts from the GEO database sets observed favourable disease outcomes in breast cancer with higher lncRNA EPB41L4A-AS2 expression. Patients with low expression lncRNA EPB41L4A-AS2 had adverse clinical outcomes (Xu et al., 2016). Lower expression of lncRNA EGOT in breast cancerous tissues was associated with larger tumour size (*p* = 0.022), lymph node metastasis (*p* = 0.020), and higher Ki-67 positivity (*p* = 0.017). A multivariate analysis suggested that a low level of lncRNA EGOT acts as an independent prognostic factor for poor survival rate in breast cancer patients (HR = 1.857, 95% CI = 1.032–3.340, *p* = 0.039) (Xu et al., 2016). Furthermore, low expression of lncFOXO1 in breast cancer tissues was associated with poorer overall survival. Functional assays demonstrated that lncFOXO1 modulates the BAP1 (BRCA-1-associated protein 1) and regulates its binding at FOXO1 promoter (Xi et al., 2017). Yang et al. (2016) demonstrated that lncRNA FGF14-AS2 was significantly down-regulated in cancer tissues having larger tumour size and more lymph node metastasis. Kaplan-Meier analysis showed that low FGF14-AS2 expression was associated with worst overall survival (Yang et al., 2016). Low relative expression of lncRNA LINC00628 in tumour tissues and breast cancer cell line had significant association with the poor prognosis and overall survival.

It has been reported that ectopic induced expression of lncRNA MAGI2-AS3 in MDA-MB-231 and MCF-7 cell lines inhibited the migration and invasiveness. The bioinformatics analysis confirmed that miRNA-342a is a direct target of lncRNA MAGI2-AS3 and its inhibition after binding with MAGI2-AS3 resulted in tumour suppressor PTEN (Phosphatase and tensin homolog) expression. Thus, the results suggested that lncRNA MAGI2-AS3 has the potential to serve as an anticancer therapeutic candidate (Du et al., 2019). Tumour suppressor lncRNA PTENP1 inhibited the proliferation and migration of breast cancer cells *via* modulating expression of cyclin A2, CDK2, p-Akt, p-p44/42 MAPK, and p-p38 MAPK cancer signalling molecules (Du et al., 2019). Similarly, PTENP1 also suppressed the miR-19b and modulated PI3K/Akt cancer signalling pathway (Shi et al., 2018). LncRNA LINC01125 exhibited an anti-proliferation effect by activation of apoptosis through PTEN/Akt/MDM2 (mouse double minute 2 homolog)/p53 cancer signalling pathway (Wan et al., 2019). Downregulation of LncRNA MALAT1 in both *in-vivo* and *in-vitro* model system induced the EMT process in cancer *via* regulation of PI3K (phosphatidylinositide-3 kinase)/Akt pathways. Therefore, MALAT1 may act as a promising therapeutic target for breast cancer metastasis *via* the PI3K-Akt pathway (Xu et al., 2015b).

A study by Yang et al. (2018b) provided new insights for treating breast cancer through the induced expression of MAGI2-AS3 and elevation of the FasR (Fas receptor) and FasL (Fas ligand) (Yang et al., 2018b). Overexpression of LINC00628 suppressed breast cancer cells proliferation, invasion and migration as well as arrested cancer cell in G0/G1 phase, upregulated caspase-3, Bax (Bcl-2-associated X), and downregulated Bcl-2 (Chen D. Q. et al., 2017). Upregulation of lncRNA CASC2 inhibited the cancer cells viability and elevated the apoptosis in cancer cells. The absence of CASC2 was related to the high expression of miR-96-5p and the downregulation of its target gene SYVN1 (Synoviolin). Thus, SYVN1 inhibited the growth and metastasis through the miR-96-5p/SYVN1 axis (Gao et al., 2018).

Similarly, the xenograft model study found downward expression of lncRNA MALAT1, which resulted in breast cancer metastasis suppression. Further analysis showed that MALAT1 inhibited the pro-metastatic transcription factor TEAD (Transcriptional enhanced associate domain) and its binding with co-activator YAP1, leading to reduced metastatic ability (Kim et al., 2018). Another study identified that tumour suppressor lncRNA-CTD-2108O9.1 inhibits metastasis by targeting LIFR (Leukemia inhibitory factor receptor) gene (Wang M. et al., 2018). Further, the lncRNA LINC00641 expression level was negatively corelated with large tumour size and lymph node metastasis. Endogenous miR-194-5p is a direct target for LINC00641 and its downregulation induced apoptosis in breast cancer cells (Wang M. et al., 2018). Xiu et al. (2019) demonstrated that induced expression of lncRNA LINC00968 negatively targeted WNT2 through HEY1 (Hes related family BHLH transcription factor with YRPW motif 1) gene regulation (Xiu et al., 2019). However, lower expression of lncRNA TUSC8 was associated with metastasis and EMT changes. The findings of another study suggested that TUSC8 inhibited breast cancer growth and metastasis *via* the miR-190b-5p/MYLIP (Myosin regulatory light chain interacting protein) axis, thus providing evidence for potential therapeutic targets for breast cancer patients (Zhao et al., 2020). On the other hand, downregulation of lncRNA FGF14-AS2 and upregulation of its target miR-205-5p indicated poor clinical outcomes (Yang Y. et al., 2019).

### 4.3 Long non-coding RNAs in early breast cancer detection

It is well known that breast cancer detection at early stage helps in the better management of disease with reduced exposer to cytotoxic chemotherapy. Several studies have identified various lncRNAs associated specifically with early breast cancer. For example, transcriptomic studies (RNA-seq) identified lncRNA LINC00885 expression in both normal and ductal carcinoma *in situ* (DCIS) breast cells (Abba et al., 2020). Expression of lncRNA BHLHE40-AS1 increases with disease progression from DCIS to invasive ductal carcinoma. Also, lncRNA BHLHE40-AS1 modulated interleukin (IL)-6/STAT3 activity and created an immune-permissive microenvironment (DeVaux et al., 2020). Overexpression of lncRNA LINC00968 was also reported at the early-stage of breast cancer. Another study demonstrated that lncRNA LINC00968 inhibited proliferation by increasing PROX1 (Prospero homeobox 1) expression through targeting miR-423-5p (Sun et al., 2019). Similarly, a lower expression of lncRNA TFAP2A-AS1 was assessed in early breast cancer patients (Zhou B. et al., 2019). Further, knockdown of lncRNA HOXA11-AS in breast cancer cell line inhibited the colony formation and arrested the cell cycle at the G0/G1 phase (Su and Hu, 2017). In addition, out of 48 lncRNAs assessed, one lncRNA (LINC01614) was highly expressed and found to have had a stronger prognostic value in early-stage breast cancer patients (Wang et al., 2019g).

### 4.4 Long non-coding RNAs in breast cancer subtypes

#### 4.4.1 Luminal

Gene expression profiling deciphered the breast cancer into four distinct molecular subtypes such as Luminal, Her2+, Her2 enriched, TNBC, and basal like. Patients with same molecular subtype responded differently to targeted therapy and showed diverse clinical outcomes. However, the exact underlying mechanism for molecular heterogeneity remains to be elucidated. Many researchers have evaluated molecular subtype specific lncRNAs expression in breast cancers and suggested its involvement in cancer molecular heterogeneity (Dastmalchi et al., 2021). Computational methods using TCGA human breast cancer data found lncRNA T-UCR overexpression and worst clinical outcomes and short survival in luminal A subtype (Marini et al., 2017). Zidan, et al. (2018) reported higher lncRNA MALAT1 expression with positive lymph node metastasis, large tumour size and proposed MALAT1 a potential prognostic candidate (ROC; 83.7% and 81.2%, sensitivity and specificity, respectively) in ER-positive breast tumour (Zidan et al., 2018). Gene expression profile study on >600 ER positive breast cancer patients, identified a set of six lncRNAs significantly correlated with overall survival in patients (Zhong et al., 2017). Li et al. (2018c) suggested that the aggressive proliferation of ER-positive breast cancer cells resulted from the higher expression of lncRNA MIAT (Li et al., 2018c).

#### 4.4.2 Her2/neu positive

Lee et al. (2017) demonstrated that induced downregulation of lncRNA snaR significantly inhibited proliferation as well migration of SK-BR3 Her2 overexpressing breast cancer cells (Lee et al., 2017). Another study showed lncRNA ES3 elevated expression in Her2-positive breast cancer samples compared to luminal A, B, and TNBC subtypes (Keshavarz et al., 2019). The lncRNA TUG1 induced higher expression in HER2-enriched invasive breast carcinoma was associated with poor survival (Gradia et al., 2017).

#### 4.4.3 Triple negative breast cancer

The use of anti-lncRNA ASBEL antago suppresses TNBC growth as a result of BTG3 (B cell translocation gene 3) gene expression restoration (Xia et al., 2017). Further, in TNBC tissues and MDA-MB-23 cells, lncRNA TP73-AS1 inhibited miR-490-3p and caused vasculogenic mimicry (VM) through upregulation of TWIST1 (Tao et al., 2018). LncRNA LRRC75A-AS1 sponged miR-380–3p and control EMT process by regulating miR-380–3p/BAALC pathway in TNBC samples (Li et al., 2020b). Inhibition of oncogenic lncRNA MALAT1 arrested TNBC cells in the *in-vivo* and *in-vitro* systems (Zuo et al., 2017). Another study found a positive correlation between lncRNAs HOST2 and CDK6 expression TNBC tissues (Lu et al., 2018b). Greater expression of LncRNA TUG1 was positively related with chemotherapy sensitivity in TNBC through inactivation WNT signalling and upregulation of NLK (Nemo-like kinase) mediated by inhibition of miR-197 (Tang et al., 2018). Presence of tumour suppressor lncRNA ZEB1-AS1 promoted cell apoptosis in TNBC tissues by stabilizing the ZEB1 mRNA *via* binding to ELAVL1 (Luo et al., 2020). Beltrán-Anaya et al. (2019) reported enhanced growth of death resistant TNBC cells absent in lncRNA KLHDC7B (Kelch domain containing 7B) (Beltrán-Anaya et al., 2019). Higher expression of lncRNA HMMR antisense RNA 1 in MDA-MB-231 and MDA-MB-468 breast cancer cells enhanced the proliferation and migration significantly (Liu et al., 2016). By assessing TCGA human breast cancer data, Mitobe et al. (2020) evaluated the overexpression of lncRNA TMPO-AS1 in basal-like breast cancer subtype. LncRNA TMPO-AS1 modulate the TGF-β and E2F signalling pathways (Mitobe et al., 2020). Another study depicted a higher lncRNA SNHG22 expression in TNBC tissues and lower expression of miR-324-3p. This observed inverse relationship caused the higher proliferation rate in TNBC *via* lncRNA SNHG22/miR-324-3p signalling pathway (Fang et al., 2020). Wang et al. showed linc-ZNF469-3 high expression in lung-metastatic LM2-4175 TNBC cells. Elevated expression of linc-ZNF469-3 promoted the lung metastasis of TNBC through miR-574-5p-ZEB1 (Zinc Finger E-Box Binding Homeobox 1) signalling axis (Wang P. S. et al., 2018). RNA immunoprecipitation confirmed the interaction between lncRNA linc003339 and miR-377-3p which positively affected TNBC proliferation and had negative effect on cell cycle arrest and apoptosis inhibition. Interaction between miR-377-3p and linc00339 mediated TNBC proliferation HOXC6 (Homeobox protein hox-C6) expression upregulation (Wang et al., 2019d). Further, blocking of lncRNA sONE resulted in high expression of downstream tumour suppressor miRNAs (miR-34a, miR-15, miR-16, and let-7a) and slowed tumour growth (Youness et al., 2019).

Long noncoding RNAs also has capacity to regulate the expression of cancer stem cell marker in TNBC. Oncogenic lncRNA DANCR induced CD44, ABCG2 (ATP Binding Cassette Subfamily G Member 2), and ALDH1 (Aldehyde dehydrogenase 1) marker’s expression (Youness et al., 2019). Similarly, lncRNA CCAT2 promoted expression of Oct4, nanog, and KLF4 genes (Kruppel-like factor 4) and growth of ALDH+ cancer stem cells in TNBC *via* targeting miR-205 (Xu et al., 2020). Microarray results observed the up-regulation of lncRNA DCST1-AS1 in TNBC tissues and cell lines and a positive correlation with poor histopathological grades. Further, a negative relation was established between lncRNA DCST1-AS1 and miR-873-5p expression and this interaction increased the expression of the CD44 marker (Tang et al., 2020). LncRNAs LINC01133 enhanced expression of pluripotency determining gene KLF4 in the TNBC targeting miR-199a-FOXP2 pathway (Tu et al., 2019).

### 4.5 Long non-coding RNAs in therapy resistance

Chemotherapy resistance is the major cause of cancer related deaths. LncRNAs have a key role in developing resistance against radiotherapy, chemotherapy, immunotherapy, and targeted therapy. Inhibition of lncRNA LINC02582 expression increased radiosensitivity miR-200c/LINC02582/CHK1 in breast cancer samples (Wang et al., 2019a). LncRNA CASC9 induced the drug-resistant breast cancer cells through the regulation of EZH2 (Jiang B. et al., 2018). Li et al. (2017c) observed four-fold higher expression of lncRNA CRALA in cisplatin poor responded breast cancer cells and its inhibition re-sensitized the cancer cells to cisplatin (Li et al., 2017c).

#### 4.5.1 Tamoxifen resistance

Tamoxifen drug is used for the treatment of ER positive breast cancer, especially in postmenopausal patients (Yao et al., 2020). Tamoxifen does two roles: first, it competes with 17β-estradiol (E2) at the receptor site and block E2; second, it binds with DNA after metabolic activation and inhibit carcinogenesis (Yao et al., 2020). However, ERα downregulation in cancer causes tamoxifen resistance. In recent years, role of non-coding RNAs in the tamoxifen resistance have been well noted. A penal of 11 lncRNAs was negatively associated with relapse-free survival (RFS) in ER-positive breast cancer patients receiving tamoxifen. The study proposed that resulted RFS might be due to deregulation of PI3K-Akt and Wnt pathway (Wang K. et al., 2018). Increased lncRNA H19 expression induced the tamoxifen and fulvestrant resistance in ETR cancer cells (Basak et al., 2018). Ozeş et al. (2017) described that inhibition of lncRNA HOTAIR sensitized the tumour cells to platinum-based chemotherapy. Inhibition of HOTAIR blocks its binding to the EZH2 and reduce NF-kβ activation and expression of its target genes such as MMP-9 and IL-6 (Özeş et al., 2017). Similarly, lncRNA ROS inhibition by use siROR sensitized breast cancer cells against tamoxifen drug. LncRNA ROS inhibition increased autophagy markers light chain 3, and beclin 1, thus, activated autophagy (Li et al., 2017b). Cai et al. (2016) investigated that lncRNA CCAT2 induced the tamoxifen resistance in MCF-7 and T47D cells (Cai et al., 2016). Furthermore, a direct relation was found in high lncRNA UCA1 expression and reduced response to tamoxifen drug. LncRNA UCA1 interacts with EZH2 and suppressed the expression of p21 through histone methylation (H3K27me3) on the p21 gene promoter. Similarly, Li et al. (2019e) concluded that lncRNA UCA1 regulated EZH2/p21 axis and PI3K/Akt signalling pathway in tamoxifen-resistant breast cancer cells (Li et al., 2019e). Additional findings revealed that tamoxifen induced lncRNA UCA1 upregulation in ER-positive breast cancer cells in a HIF-1α (Hypoxia-inducible factor-1alpha) dependent manner, and thus enhanced tamoxifen resistance (Li et al., 2016). LncRNA H19 induced autophagy activation *via* the H19/SAHH/DNMT3ß (DNA (cytosine-5)-methyltransferase 3 beta) axis, contributed to tamoxifen resistance in breast cancer (Wang et al., 2019b). Downregulated lncRNA ROR supressed EMT and sensitized MDA-MB-231 cells to tamoxifen. miR-205 is a direct target of lncRNA ROR, which subsequently affects ZEB1 and ZEB2 (Zhang H. Y. et al., 2017).

#### 4.5.2 Doxorubicin resistance

Doxorubicin is a Streptomyces peucetius bacterium derived antibiotic molecule, being used as a chemotherapeutic agent since the 1960s. It is a member of anthracycline group of chemotherapeutic agents (Thorn et al., 2011). Doxorubicin can inhibit cancer cell growth by following mechanism: 1) intercalation with DNA that disrupt topoisomerase-II-mediated DNA repair, 2) generation of free radicals which damage cell membrane, DNA, and proteins (Thorn et al., 2011). Still, cancer cell can overcome the anti-tumour effects of doxorubicin. Among the other therapy resistance mechanisms, regulation of long non-coding RNAs is one of the recently reported mechanism. For instance, elevated lncRNA LINP1 in breast cancer was related to doxorubicin & fluorouracil chemoresistance and its knockout caused G1-phase cell cycle arrest and activation of apoptosis (Liang et al., 2018a). A study by Wang et al. found increased lncRNA H19 expression in doxorubicin resistant breast cancer and its suppression significantly lowered doxorubicin resistance (Wang X. et al., 2020).

#### 4.5.3 Trastuzumab resistance

Trastuzumab is an FDA approved humanized monoclonal antibody used as targeted therapy in Her-2 positive breast cancer. Mechanistically, trastuzumab binds to an extracellular domain of ERBB2 receptor and inhibit its homodimerization, thereby preventing ERBB2-mediated signaling (Vu and Claret, 2012). Trastuzumab can also degradation degrade ERBB2 receptor, mediate antibody-dependent cellular cytotoxicity (ADCC), and interfere with MAPK and PI3K/Akt signaling pathways (Vu and Claret, 2012). However, cancer cell can mediate the expression of lnc RNAs and thereby survive against the cytotoxic effect of trastuzumab. According to Dong et al., lncRNA SNHG14 induced trastuzumab (ERBB2/HER2 antibody) resistance in HER2+ breast cancer tissues. Mechanistically, lncRNA SNHG14 regulated PABPC1 (Polyadenylate-binding protein 1) gene through H3K27 acetylation and hence activation of Nrf2 (Nuclear factor erythroid 2-related factor 2) signalling pathway (Dong et al., 2018). Zhu et al. (2018) showed knockout lncRNA UCA1 in SKBR-3 breast cancer cell, resulting in trastuzumab sensitivity *via* lncRNA UCA1/miR-18a/YAP1 axis (Zhu et al., 2018). High expression of lncRNA HOTAIR in SK-BR-3-TR trastuzumab-resistant breast cancer cell line induced EMT confirmed by dysregulation of marker i.e., TGF-β, Snail, Vimentin, and E-cadherin (Chen et al., 2019).

#### 4.5.4 Paclitaxel resistance

Paclitaxel, is a taxane and inhibits the cancer cell growth by regulating microtubule stabilising ability, arrests cell in the G2/M-phase of the cell cycle, eventually push the cancer cell to undergo apoptosis (Kampan et al., 2015). Still cancer cell acquired the resistance against paclitaxel by controlling the expression of lnc RNAs. For example, higher expression was found in lncRNA FTH1P3 showing paclitaxel resistance in MCF-7/PTX and MDA-MB-231/PTX cells. In xenograft mice, lncRNA FTH1P3 targeted miRNA-206 and upregulated ABCB1 (ATP Binding Cassette Subfamily B Member 1) protein (Wang R. et al., 2018). Li et al. (2017) measured four-fold higher expression of lncRNA CRALA in paclitaxel poor responded breast cancer cells (Li et al., 2017c). Paclitaxel-resistant breast cancer tissue and cell line had downregulation of lncRNA which led to upregulation of miR-18b-5p and inhibited DOCK4 (Dedicator of cytokinesis protein 4) (Wang Y. Y. et al., 2019). Zheng et al. reported that lncRNA CASC2 activated paclitaxel resistance in breast cancer through regulation of miR-18a-5p/CDK19 (Zheng P. et al., 2019).

## 5 Long noncoding RNA in cancer stem cell maintenance

Stem cells population in the tumour milieu is related with tumour maintenance and therapy failure. Several studies determined lncRNAs can regulate the expression of stem cell markers. Such as, lncRNA H19 upregulate the Sox4 in cancer cell *via* downregulation of miR-138 (Si et al., 2019). Upregulation of lncRNA MIAT in ER/PR+, HER2-, and TNBC samples significantly modulated Oct4 (octamer-binding transcription factor 4) mRNA levels (Almnaseer and Mourtada-Maarabouni, 2018). Another study showed that lncRNA ES1 upregulation in both high-grade and p53-mutated breast tumour tissues enhanced the Oct4/Sox2 makers by regulating the Oct4/Sox2/miR-302/miR-106b axis (Keshavarz and Asadi, 2019). Further, overexpressed lncRNA FOXD2-AS1 regulated the expression of stem cell markers (Oct4, Nanog, and SOX2) *via* FOXD2-AS1/miR-150-5p axis (Jiang et al., 2019). The loss-of functional study indicated that FEZF1-AS1 knockout reduced the CD44+/CD24- rate, mammosphere-forming ability, and stem factors i.e., Nanog, Oct4, and SOX2 (Zhang et al., 2018). The study by Lu et al. (2018a) also revealed that LINC00511 promoted stem factors Oct4, Nanog, and Sox2 expression (Lu et al., 2018a). Further, expression of lncRNA SOX2OT modulates Sox2 in ER positive and negative breast cancer samples (Askarian-Amiri et al., 2014). For instance, lncCUEDC1 has demonstrated as a negatively regulator for phenotype and biological functions of breast cancer stem cells (BCSCs) by inhibiting NANOG (Zhang F. et al., 2020). Also, study demonstrated that MALAT-1 affects the stem cell-like phenotypes in breast cancer cells through regulation of Sox-2 (Zeng et al., 2018). The lncRNA NRAD1 contributed to gene expression changes which were associated with cancer stem cell by involving ALDH1A3 (Vidovic et al., 2020). Another study found that lnc030 increased cholesterol synthesis through cooperates with poly (rC) binding protein 2 (PCBP2) and governs BCSC stemness (Qin et al., 2021). In addition, lncRNA KB-1980E6.3 maintains the stemness of BCSCs through lncRNA KB-1980E6.3/IGF2BP1/c-Myc axis (Zhu et al., 2021). Moreover, lncTHOR in TNBC compared to that in luminal A and luminal B molecular subtype, facilitates stemness through activating ß-catenin signaling (Wang B. et al., 2020). Also, LncCCAT2 in TNBC through upregulating OCT4-PG1 expression and activating Notch signaling, controlled aggressiveness of breast cancer stem cells (Xu et al., 2020). Furthermore, LINC01133 also determined as regulator of the pluripotency-determining gene Kruppel-Like Factor 4 (KLF4) in TNBC (Tu et al., 2019). In an *in-vitro* model, overexpression of SOX21-AS1 enhanced the proliferation, migration and invasion of CSC-MCF-7 cells *via* inhibiting the Hippo pathway (Li et al., 2021). A study highlighted that LINC00261 can adsorb miR-550a-3p to modulate SDPR, and thus inhibited migration and invasion of CD44^+^/CD24-/low BCSCs, exerting a potential effect on therapy (Li and Wu, 2021). Upregulation of pluripotent lncRNA ES3 was significantly upregulated in Her-2 positive breast tumours and may contribute to breast cancer proliferation as a downstream target of Her-2 (Keshavarz et al., 2019). Thus, significant work established lncRNAs signature in BCSCs and these findings assess us with evidence to explore further functionalities of lncRNAs in BCSCs and provide a novel therapeutic strategy for breast cancer (Ge et al., 2020).

## 6 Long non-coding RNAs as non-invasive biomarkers

Invasive biopsy procedures are painful interventions. These procedures also induced certain anatomical and structural deformities. Liquid biopsy is an alternative painless option that can be used for diagnostic purposes. Liquid biopsy includes taking different body fluids, most commonly blood or serum for identification of diagnostic and therapeutic biomarkers in the patients. Researchers have found different lncRNAs in the blood or serum of breast cancer patients. Therefore, lncRNAs may serve as non-invasive biomarkers. A number of studies have identified the expression of lncRNA HOTAIR in the blood of breast cancer patients where it was associated with the high expression of ERBB2 (Receptor tyrosine-protein kinase) (Wang Y. L. et al., 2019). Bermejo et al. (2019) observed hypermethylated lncRNA LINC00299 in TNBC breast cancer patients peripheral blood compared to the normal healthy controls. Levels of lncRNA LINC00310 were significantly enhanced in the serum of breast cancer patients. Receiver operating characteristic (ROC) curve analysis indicated that lncRNA LINC00310 had a powerful capability of distinguishing breast cancer patients from healthy individuals (area under curve 0.828) (Li et al., 2018a). Another study revealed higher amounts of lncRNAs H19, HOTAIR, and RP11-445H22.4 in the plasma of breast cancer patients compared to the normal healthy controls (Jiao et al., 2018).

## 7 Conclusion and future perspectives

Long ncRNAs like H19 and XIST were discovered in the pre-genomic era, but were not fully characterized and explored until the early 2000s (Jarroux et al., 2017). Invention and improvement in DNA sequencing or high throughput RNA sequencing facilitated the discovery of non-coding DNA such as lncRNAs genes sequencing with some functional role and their involvement in the various pathological and disease conditions. The profiling of differential expression of lncRNAs with massive parallel RNAseq and single-cell RNAseq technologies revealing its significant impact on breast cancer biology. Using these sophisticated technologies, different scientific groups around the world discovered a range of lncRNAs genes and profiled them in different types of disease, including cancer. Bioinformatic tools and gene enrichment analysis correlated these lncRNAs with different signalling pathways and highlighted their diagnostic virtues and treatment monitoring importance. Despite this huge information on lncRNAs and their identification in disease conditions, their clinical use is still limited. The main reason is the non-reproducibility of results obtained in different labs. Unfortunately, the results obtained from several studies do not match even though they have been conducted on a single disease condition. Perhaps, non-reproducibility of results is due to the variations in sensitivity of different techniques and protocols used in sample collection under different conditions. The experimental and clinical evidence provided in this comprehensive review supports the use of lncRNAs as a prognostic and predictive biomarker in breast cancer patients even in respective molecular subtypes of breast cancer. Since the clinical importance of lncRNAs is now getting established, it will help to reduce the non-reproducibility and enhance the accuracy of results in breast cancer patients.
