# Supplementary material for: Coding roles of long non-coding RNAs in breast cancer: Emerging molecular diagnostic biomarkers and potential therapeutic targets with special reference to chemotherapy resistance
Source: Front Genet. 2023 Jan 6;13:993687. doi: 10.3389/fgene.2022.993687 (PMC9852779; doi:10.3389/fgene.2022.993687)
Supplement: Supplementary file 1 [file Table1.docx]

**Supplementary Table 1: Summary of functions, targets, and experimental models used for studying the upregulated long noncoding RNAs in different in vivo and in vitro investigations.**

| **LncRNA** | **Status** | **Target** | **Consequences** | **In vivo** | **In vitro** | **Technique** | **Ref** |
| --- | --- | --- | --- | --- | --- | --- | --- |
| LncRNA CCAT2 | ↑ | TGF-β signaling pathway | Metastasis | Breast cancer tissue | MDA-MB-231  HCC1937  LCC9 &  MCF-7 | qRT-PCR | (Wu et al., 2017) |
| LncRNA NKILA | ↑ | NF-kβ/Ikβ | Inflammation stimulated breast epithelial cells | Breast cancer tissue | - | qRT-PCR | [26] |
| LncRNA DANCR | ↑ | EZH2 | Worse prognosis | TNBC  tissues | MDA-MB-231 | qRT-PCR | (Sha et al., 2017) |
|  | ↑ | EZH2 | Worse prognosis | ER/PR/Her2 (+/-) breast cancer tissues &  male Balb/c nu/nu | MDA-MB-231  MDA-MB-468  MCF7 &  T47D | qRT-PCR | (Zhang et al., 2020b) |
| LncRNA UCA1 | ↑ | miRNA-143 | Modulate cell growth and apoptosis | ER/PR/Her2 (+/-) breast cancer tissues | HEK 293T &  MDA-MB-231 | qRT-PCR | (Tuo et al., 2015) |
|  | ↑ | miRNA-18a | Herceptin  resistant | - | SKBR-3 | qRT-PCR | (Zhu et al., 2018) |
|  | ↑ | - | Promote carcinogenesis | - | MCF-7 | qRT-PCR | (Chen et al., 2015) |
|  | ↑ | EZH2 | Regulate the PI3K/Akt pathway, &  Tamoxifen resistance | Breast cancer tissues | MCF‑7  T47D  LCC2 &  LCC9 | qRT-PCR | ‑(Li et al., 2019e) |
|  | ↑ | miR-18a | Tamoxifen resistance by regulating HIF-1α gene expression | - | MCF-7  BT474  LCC2 &  LCC9 | qRT-PCR | (Li et al., 2016) |
|  | ↑ | hnRNP I | Promote cell growth by inhibition of p27 | Female nude mice | MDA-MB-231  HCT-116 p53-WT  HCT-116 p53-null &  MCF-7 | qRT-PCR | (Huang et al., 2014) |
| LncRNA YIYA | ↑ | CDK6 | Promote  glycolysis | Breast cancer tissues | MDA-MB-231 MCF7  BT474 &  HEK293T | RNAscope assay,  IHC | (Xing et al., 2018) |
| LncRNA ROR | ↑ | Light chain 3,  Beclin 1 | Autophagy | Breast cancer tissues &  nude mice | MCF7, BT474 MDA-MB-435 &  MDA-MB-231 | qRT-PCR | (Li et al., 2017b) |
|  | ↑ | - | Regulate the TGF-β signaling pathway | Female  Nude &  Breast cancer tissues | MDA-MB-231 &  MCF-7 | qRT-PCR | (Hou et al., 2018) |
|  | ↑ | miR-205 | Tamoxifen resistance | - | MDA-MB-231  MCF10A &  MCF7 | qRT-PCR | (Zhang et al., 2017b) |
| LncRNA PRNCR1 | ↑ | - | Poor  prognosis | ER/PR/Her2 (+/-) breast cancer tissues | MDA‑MB‑231  BT‑549  MCF‑7 &  SK‑BR‑3 | qRT-PCR | ‑(Guo et al., 2019) |
| LncRNA NONHSAT028712 | ↑ | HSP90 | Cancer cell growth and metastasis through CDK2 activation | Breast cancer tissues &  BALB/c mice | MCF-7/ADM  MCF-7  MDA-MB-231 | qRT-PCR | (Cui et al., 2020) |
|  | ↑ | - | Poor prognosis | Her2+ &  TNBC tissues | MDA-MB-231  MCF | qRT-PCR | (Jadaliha et al., 2016) |
|  | ↑ | TEAD | Suppress metastasis | Mice | HEK293FT  MDA-MB-231 | qRT-PCR | (Kim et al., 2018) |
|  | ↑ | miR-129-5p | Poor prognosis | TNBC tissues | MDA-MB-231  MDA-MB-453 MCF-7  TB-549  BT-474 | qRT-PCR | (Zuo et al., 2017) |
|  | ↑ | - | Poor prognosis | ER/PR/Her2 (+/-) breast cancer tissues | - | qRT-PCR | (Zidan et al., 2018) |
|  | ↑ | miR-339-5p | Poor prognosis &  Regulate BLCAP | Breast cancer sample data from TCGA &  ER/PR/Her2 (+/-) breast cancer tissues | MCF7 | qRT-PCR | (Zheng et al., 2019b) |
| LncRNA CCAT1 | ↑ | - | Poor prognosis | ER/PR/Her2 (+/-) breast cancer tissues | - | qRT-PCR | (Zhang et al., 2015) |
| LncRNA Z38 | ↑ | - | Poor prognosis | ER/PR/Her2 (+/-) breast cancer tissues | - | qRT-PCR | (Nie et al., 2018) |
|  | ↑ | - | Increase proliferation | - | MDA-MB-231  MDA-MB-453 MCF-7  HBL-100  BT20  T47D | SSH | (Deng et al., 2016) |
| LncRNA FTH1P3 | ↑ | miRNA-206 | Paclitaxel resistance by upregulating ABCB1 gene expression | Breast cancer tissues &  male BALB/c  nude mice | MCF-7/PTX  MDA-MB- 231/PTX | qRT-PCR | (Wang et al., 2018e) |
| LncRNA LINC00310 | ↑ | c-Myc | Increase Proliferation | Breast cancer tissues &  nude mouse xenograft | MDA-MB-231  LM-4142  MCF-7 | qRT-PCR  CRISPR/Cas9 system | (Li et al., 2018a) |
| LncRNA SNHG14 | ↑ | PABPC1 | Trastuzumab resistance, &  Activate Nrf2 signaling pathway | Breast cancer tissues | SKBR‐3  BT-474  SKBR‐3/Tr  BT474/Tr | qRT-PCR | (Dong et al., 2018) |
| LncRNA SNHG7 | ↑ | miRNA-381 | Poor prognosis | Breast cancer tissues | MDA-MB-231  MDA-MB-468  ZR-75-1  HCC-1973 | qRT-PCR | (Gao and Zhou, 2019) |
| LncRNA LINC00617 | ↑ | Sox2 | EMT | ER/PR/Her2 (+/-) breast cancer tissues,  Mouse | MDA-MB-468  MCF-7 | qRT-PCR | (Li et al., 2017a) |
| LncRNA TUG1 | ↑ | - | Poor prognosis | ER/PR/Her2 (+/-) breast cancer tissues | MDA-MB-231  MDA-MB-436  T-47D  MCF-7 | qRT-PCR | (Fan et al., 2017) |
|  | ↑ | miR-197 | Inactivate Wnt signaling,  Induce cisplatin resistance | TNBC cancer tissues | MDA-MB-231  BT549  MCF7  T47D | qRT-PCR | (Tang et al., 2018) |
|  | ↑ | - | Inhibit proliferation,  & EMT,  Increase apoptosis, autophagy,  ER stress, regulate  Akt/mTOR pathway, &  p38 MAPK/Erk  signaling | - | MCF-7 | qRT-PCR | (Huang Y et al., 2018) |
| LncRNA LINP1 | ↑ | - | Increase proliferation, metastasis,  doxorubicin & fluorouracil resistance | ER/PR/Her2 (+/-) breast  cancer tissues | MDA-MB-231 MDA-MB-468  MCF-7 | qRT-PCR | (Liang et al., 2018a) |
|  | ↑ | - | Poor prognosis | ER/PR/Her2 (+/-) breast cancer tissues | - | qRT-PCR | (Liu et al., 2018) |
| LncRNA LINC00472 | ↑ | - | Poor prognosis | ER/PR (+/-) breast cancer tissues | MCF-7  SKBR-3 | qRT-PCR  microarray | (Shen et al., 2015) |
| LncRNA EPB41L4A-AS2 | ↑ | - | Good prognosis | ER/PR/Her2 (+/-) breast cancer tissues | MDA-MB-231 | qRT-PCR  Microarray | (Xu et al., 2016) |
| LncRNA HOTTIP | ↑ | - | Poor prognosis | ER/PR/Her2 (+/-) breast cancer tissues | - | qRT-PCR  microarray | (Yang et al., 2017) |
| LncRNA T-UCR | ↑ | - | Poor prognosis | ER/PR/Her2 (+/-) breast cancer tissues | MCF-7 | qRT-PCR  Microarray | (Marini et al., 2017) |
| LncRNA GAS5 | ↑ | - | Activate Apoptosis | - | MDA-MB-231  MCF7  T-47D | qRT-PCR | (Pickard and Williams, 2014) |
| LncRNA U79277, LncRNA AK024118, LncRNA BC040204,  LncRNA AK000974 | ↑ | - | Increase metastasis | Breast cancer samples data from GSE20685,  GSE21653, GSE12276, &  GSE42568 | - | qRT-PCR  Microarray | (Meng et al., 2014) |
| LncRNA LUCAT1 | ↑ | miR-5582-3p | Regulate Wnt/β-catenin pathway | Sample from TCGA-BRCA database, &  female BALB/c (nu/nu) mice | MCF-10A  MCF-7  T47D | qRT-PCR  FISH | (Zheng et al., 2019a) |
| LncRNA NEAT1 | ↑ | - | Regulate apoptosis,  cell cycle,  stem cell features | Blood from ER/PR/Her2 (+/-) cancer patients | MDA-MB-231 | qRT-PCR | (Shin et al., 2019) |
|  | ↑ | miR-146b-5p | Increase proliferation, migration invasion, &  EMT | ER/PR/Her2 (+/-) breast cancer tissues | MDA-MB-231 MDA-MB-453  BT474  MCF-7  SKBR-3 | qRT-PCR | (Li et al., 2020a) |
| LncRNA HOTAIR | ↑ | EZH2 | Reduce activation & expression of NF-kβ,  MMP-9  IL-6 gene expression,  Sensitize cells to platinum | - | MDA-MB-231  MCF-7  SKBR3 | qRT-PCR | (Özeş et al., 2017) |
|  | ↑ | - | Increase ERBB2 gene expression | Blood from ER/PR/Her2 (+/-) cancer patients | MDA-MB-231  MCF-7 | qRT-PCR | (Wang et al., 2019f) |
|  | ↑ | - | Invasion and metastasis by upregulation of CHST15 gene expression | - | MDA-MB-231  MDA-MB-468 | ISH | (Liu et al., 2019) |
|  | ↑ | miR-206 | Regulate Bcl-w gene expression | Breast cancer tissues | MCF-7  T47D | qRT-PCR | (Ding et al., 2017) |
|  | ↑ | - | Trastuzumab  resistance | Nude mice | SK-BR-3 | qRT-PCR | (Chen et al., 2019) |
| LncRNA MIAT | ↑ | - | Regulate cell cycle | ER/PR (+/-) breast cancer tissues | MCF-7 | qRT-PCR | (Li et al., 2018c) |
|  | ↑ | - | Increase OCT4 gene expression, &  Inhibit apoptosis | - | MDA-MB-231  MCF7  Hs58T | Tissue Scan TM Breast Cancer cDNA Arrays  qRT-PCR | (Almnaseer and Mourtada-Maarabouni, 2018) |
| LncRNA TP73-AS1 | ↑ | miR-490-3p | Endothelial transition | TNBC tissues | MDA-MB-231 | QRT-PCR | (Tao et al., 2018) |
| LncRNA LINC00473 | ↑ | miR-198 | Regulate MAPK1 gene expression | ER/PR/Her2 (+/-) breast cancer tissues &  NOD/SCID mice | MDA-MB-231 MCF-7  SK-BR-3  MDA-MB-453 | qRT-PCR,  ISH,  FISH | (Niu et al., 2019) |
|  | ↑ | miR-497 | Poor prognosis | ER/PR/Her2 (+/-) breast cancer tissues | MDAMB-231  MDA-MB-453 MCF-7  MDA-MB-468 | qRT-PCR | (Jiao et al., 2018) |
| LncRNA LINC01296 | ↑ | - | Poor prognosis | ER/PR/Her2 (+/-) breast cancer tissues &  BALB/c nude mice | MDA-MB-231 SKBR3  BT-20  MCF7  T47D  MDA-MB-436 | qRT-PCR | (Jiang et al., 2018c) |
| LncRNA 91H | ↑ | IGF2 | Increase proliferation | ER/PR/Her2 (+/-) breast cancer tissues &  female SCID mice | MDA-MB-231  T47D | qRT-PCR | (Vennin et al., 2017) |
| LncRNA ASBEL | ↑ | BTG3 | Increase proliferation | - | MDA-MB-231 | qRT-PCR | (Xia et al., 2017) |
| LncRNA DLX6-AS1 | ↑ | miR-505-3p | Regulate RUNX2 gene expression | ER/PR/Her2 (+/-) breast cancer tissues | MDA-MB-231  BT-474 | qRT-PCR | (Zhao et al., 2019b) |
| LncRNA RNA MIF-AS1 | ↑ | miR-1249-3p | Regulate HOXB8 gene expression &  EMT | ER/PR/Her2 (+/-) breast cancer tissues | MDA-MB-231 MDA-MB-468  MCF-7 | qRT-PCR | (Ding et al., 2019) |
| LncRNA ST8SIA6-AS1 | ↑ | miR-4252  NONO,  QKI, &  RBMX | Increase Proliferation,  Invasion, &  migration | Breast cancer tissues | MDA-MB-453  MDA-MB-231 MDA-MB-468  MCF-7  T47D  Hs-578T  HBL-100 MCF10A | Microarray  qRT-PCR | (Chen et al., 2020b) |
| LncRNA SNHG6 | ↑ | miR-26a-5p | Upregulate MAPK6 gene expression | ER/PR/Her2 (+/-) breast cancer  tissues | MDA-MB-231  MDA-MB-468  MCF-10 A  BT-474  ZR-75-30  T-47D | qRT-PCR | (Lv et al., 2019) |
| LncRNA linc01561 | ↑ | miR-145-5p | Upregulate MMP-11 gene expression | Breast cancer sample data from TCGA | MCF-7  BT-20  ZR-75-1  MX-1 | qRT-PCR | (Jiang et al., 2018d) |
| LncRNA LOXL1-AS1 | ↑ | miR-708-5p | Upregulate NF-kβ expression | ER/PR/Her2 (+/-) breast cancer  tissues &  female BALB/c nude mice | MDA-MB-231 MDA-MB-468  T47D  MCF-7 | qRT-PCR | (Dong et al., 2020) |
| LncRNA H19 | ↑ | - | Doxorubicin resistance | - | MDA‐MB‐231  MCF‐7 | qRT-PCR | (Luo et al., 2020) |
|  | ↑ | - | Tamoxifen & Fulvestrant resistance,  Activate Notch and c-MET signaling | - | LCC2  LCC9 | qRT-PCR | (Basak et al., 2018) |
|  | ↑ | miR-138 | Poor prognosis,  Increase EMT,  Upregulate SOX4 gene expression | Breast cancer tissues | MDA-MB-231  LCC9  LCC2  MCF-7 | qRT-PCR | (Si et al., 2019) |
|  | ↑ | - | Inhibit proliferation,  & EMT,  Increase apoptosis, autophagy,  ER stress, regulate  Akt/mTOR pathway, &  p38 MAPK/Erk  signaling | - | MCF-7 | qRT-PCR | (Huang Y et al., 2018) |
|  | ↑ | miR-152 | Regulate DNMT1 gene expression | Breast cancer tissues | MDA-MB-231  MCF-7 | qRT-PCR | (Li et al., 2017d) |
|  | ↑ | miR‐93‐5p | Regulate  STAT3 gene expression | - | MDA‐MB‐231  HEK293T  MCF‐7 | qRT-PCR | (Li et al., 2019a) |
|  | ↑ | - | Autophagy activation via H19/SAHH/DNMT3B axis &  tamoxifen resistance | MCF7/TAMR-Tet-shH19 xenografts | MCF7  MCF7/TAM | qRT-PCR | (Wang et al., 2019b) |
| LncRNA BANCR | ↑ | - | Inhibit Apoptosis, &  Induce EMT | ER/PR/Her2 (+/-) breast cancer tissues | MDA-MB-231  MCF-7  SKBR-3  BT-20 | qRT-PCR | (Jiang et al., 2018b) |
| LncRNA CRALA | ↑ | - | Chemoresistance | ER/PR/Her2 (+/-) breast cancer tissues | MDA-MB-231  MDA-MB-231P  MDA-MB-231C | qRT-PCR | (Li et al., 2017c) |
| LncRNA TP73-AS1 | ↑ | miRNA-125a-3p | Inhibit apoptosis,  increase migration, invasion | ER/PR/Her2 (+/-) breast cancer  cancer tissues | MCF-10A  HCC-70  MB231 | qRT-PCR | (Liu et al., 2020) |
| LncRNA KLHDC7B | ↑ | KLHDC7B | Inhibit migration and invasion | TNBC tissues | MDA-MB-468 MDA-MB-231  MCF10A  BT-20  Hs578-T  MCF-7 HCC1187 | qRT-PCR | (Beltrán-Anaya et al., 2019) |
| LncRNA H19, LncRNA HOTAIR,  LncRNA RP11-445H22.4 | ↑ | - | Increase proliferation | Plasma form Breast cancer patients | - | qRT-PCR | (Jiao et al., 2018) |
| LncRNA CCAT2 | ↑ | miR-205 | Increase OCT4,  Nanog,  KLF4,  ALDH+ gene expression &  Activate Notch signaling | TNBC tissues | - | qRT-PCR | (Xu et al., 2020) |
|  | ↑ | - | Tamoxifen resistance | - | MCF-7  T47D | qRT-PCR | (Cai et al., 2016) |
|  | ↑ | - | Inhibit proliferation,  & EMT,  Increase apoptosis, autophagy,  ER stress, regulate  Akt/mTOR pathway, &  p38 MAPK/Erk  signaling | - | MCF-7 | qRT-PCR | (Huang Y et al., 2018) |
| LncRNA snaR | ↑ | - | Increase migration | - | MDA-MB-231  SK-BR3 | qRT-PCR | (Lee et al., 2017) |
| LncRNA LINC00673 | ↑ | B7-H6 | Increase Metastasis &  EMT | ER/PR (+/-) breast cancer tissues | MDA-MB-231  MDA-MB-468  BT-549  MCF-7  MCF-10A | qRT-PCR | (Xia et al., 2018) |
| LncRNA CRNDE | ↑ | miR-136 | Poor prognosis,  Upregulate β-catenin,  c-myc,  cyclin D1 gene expression,  regulate Wnt/β-catenin signaling pathway | ER/PR/Her2 (+/-) breast cancer tissues | MDA-MB-231 MDA-MB-468 HBL-100  MCF-7  BT-549 | qRT-PCR | (Huan et al., 2017) |
| LncRNA RHPN1-AS1 | ↑ | - | Poor prognosis | Breast cancer sample data from TCGA &  breast cancer tissues | - | qRT-PCR,  Microarray,  FISH | (Zheng et al., 2019d) |
| LncRNA HMMR-ASI | ↑ | HMMR | Poor prognosis | ER/PR/Her2 (+/-) breast cancer tissues | MDA-MB-231  MDA-MB-468 | qRT-PCR | (Liu et al., 2016) |
| LncRNA LINC00299 | ↑ | - | Diagnostic marker | TNBC tissues | - | qRT-PCR | (Bermejo et al., 2019) |
| LncRNA TMPO-AS1 | ↑ | - | Increase proliferation, &  migration | Female NOD/SCID mice &  breast cancer sample data TCGA | MDA-MB-231  MDA-MB-468 | Microarray,  qRT-PCR | (Mitobe et al., 2020) |
| APOC1P1-3 | ↑ | Tubulin | Decrease α-tubulin  acetylation, inactivate caspase-3, | Breast cancer samples data from GSE80266 | - | Microarray,  qRT-PCR | (Liao et al., 2016) |
| LncRNA LINC00707 | ↑ | miR-876 | Increase proliferation, migration, invasion | ER/PR/Her2 (+/-) breast cancer tissues | MDA-MB-231  MCF-7  BT-474  SKBR-3 | qRT-PCR | (Li et al., 2019c) |
| LncRNA Linc00460 | ↑ | miR-489-5p | Lymphatic metastasis &  activate FGF7-AKT pathway | Breast cancer sample data from GSE20711 & GSE6532 | MDA-MB-231 MCF-7  BT-474  BT-549 | qRT-PCR | (Zhu et al., 2019b) |
| LncRNA AK058003 | ↑ | SNCG | Promote proliferation, invasion and migration | ER/PR/Her2 (+/-) breast cancer tissues | MCF‑7 | qRT-PCR | (He and Wang, 2015) |
| LncRNA EZR‑AS1 | ↑ | β‑catenin | Activate Wnt/β‑catenin pathway | Breast cancer tissue | MDA‑MB‑231 MDA‑MB‑468 MCF‑10A  SKBR‑3 | qRT-PCR | (Bai et al., 2018) |
| LncRNA ES1 | ↑ | - | Upregulate Oct4,  Sox2 gene expression,  miR-302,  miR-106b expression | Breast cancer tissue | MDA-MB-231  SKBR-3 | qRT-PCR | (Keshavarz and Asadi, 2019) |
| LncRNA DICER1-AS1 | ↑ | - | Inhibit proliferation,  & EMT,  Increase apoptosis, autophagy,  ER stress, regulate  Akt/mTOR pathway, &  p38 MAPK/Erk  signaling | - | MCF-7 | qRT-PCR | (Huang Y et al., 2018) |
| LncRNA ANRIL | ↑ | POSTN | Cancer progression | ER/PR/Her2 (+/-) breast cancer tissues, | MDA-MB-231  MCF-7 | FISH | (Mehta-Mujoo et al., 2019) |
| LncRNA CASC9 | ↑ | EZH2 | Doxorubicin resistance | ER/PR/Her2 (+/-) breast cancer tissues, | MCF‑7 (HTB‑22™) MCF‑7/doxorubicin (DOX) | qRT-PCR | (Jiang et al., 2018a) |
| LncRNA HOST2 | ↑ | let-7b | Increase CDK6 gene expression,  proliferation | ER/PR/Her2 (+/-) breast cancer tissues | MDA‑MB‑231  MDA‑MB‑468 | qRT-PCR | (Lu et al., 2018b) |
| LncRNA FOXD2‑AS1 | ↑ | - | Tumourigenesis through FOXD2‑AS1/S100A1/Hippo axis | Breast cancer sample data from TCGA dataset &  male  BALB/c nude mice | MDA‑MB‑468 MDA‑MB‑453  MCF‑7  BT‑549 | qRT-PCR | (Huang and Xue, 2020) |
| LncRNA LINC00885 | ↑ | - | Poor prognosis, Regulate expression of EREG,  c-Myc, &  CCND1 genes expression | Breast cancer tissues | T47D  MCF7 | RNA-Seq,  qRT-PCR | (Abba et al., 2020) |
| LncRNA FOXD2‑AS1 | ↑ | miR‑150‑5p | Regulate stem cell markers; Nanog,  Oct4,  SOX2,  EMT markers; N‑cadherin, E‑cadherin, vimentin genes expression | ER/PR/Her2 (+/-) breast cancer tissues | MDA‑MB‑231 MDA‑MB‑453  MDA‑MB‑468  MCF‑7 | qRT-PCR | ‑‑‑‑‑‑‑(Jiang et al., 2019) |
| LncRNA GHSROS | ↑ | - | Increase  migration | - | MDA‑MB‑231  MDA‑MB‑468  MDA‑MB‑453 | qRT-PCR | (Thomas et al., 2019) |
| LncRNA UASR1 | ↑ | - | Regulate pAKT, pTSC2,  p4EBP1, pp70S6K &  AKT/mTOR pathway | Breast cancer tissues | MDA-MB‑231  MCF-7 | qRT-PCR | (Cao et al., 2019) |
| LncRNA DCST1-AS1 | ↑ | miR-873-5p | Regulate IGF2BP1, Myc,  CD44, &  LEF1 genes expression | - | MDA-MB-231  BT-549 | Microarray,  FISH,  qRT-PCR | (Tang et al., 2020) |
| LncRNA BHLHE40‐AS1 | ↑ | - | Modulate IL‐6/STAT3  signaling | - | MCF10ApBABE  MCF10ABHLHE40‐AS1 | Microarray,  FISH,  qRT-PCR | (DeVaux et al., 2020) |
| LncRNA ES3 | ↑ | - | Increase stem cell markers | ER/PR/Her2 (+/-) breast cancer tissues, | MDA-MB‐231  MDA‐MB‐435 MDA‐MB‐231  MDA‐MB‐468  SKBR‐3  MCF7 | qRT-PCR | (Keshavarz et al., 2019) |
| LncRNA HOXD‐AS1 | ↑ | miR‐421 | Regulate,  EMT by HOXD‐AS1/miR‐421/SOX4 axis | ER/PR/Her2 (+/-) breast cancer tissues, | MCF‐7 | qRT-PCR | (Li et al., 2019d) |
| LncRNA LINC00968 | ↑ | miR-423-5p | Increase proliferation,  migration and tube formation | ER/PR/Her2 (+/-) breast cancer tissues, TCGA | MDA-MB-231 BT-20  MCF-7  T-47D | Microarray,  qRT-PCR | (Sun et al., 2019) |
| LncRNA LINC00511 | ↑ | miR-185-3p | Increase Oct4, Nanog, & SOX2 genes expression | Xenograft mice &  ER/PR/Her2 (+/-) breast cancer tissues, | MDA-MB-468  MDA-MB-231  MDA-MB-453  MCF-7 | qRT-PCR | (Lu et al., 2018a) |
| LncRNA HOXA11‑AS | ↑ | - | Increase proliferation, metastasis,  Regulate cell cycle | ER/PR/Her2 (+/-) breast cancer tissues | MDA‑MB‑231  MDA‑MB‑436 MCF7  T47D | qRT-PCR | (Su and Hu, 2017) |
| LncRNA PRNCR1‑2 | ↑ | - | Increase CHK2 phosphorylation and decrease AKT phosphorylation | Breast cancer tissues | MDA‑MB‑468 MDA‑MB‑231MMCF‑7 | qRT-PCR | (Pang et al., 2019) |
| LncRNA PTENP1 | ↑ | - | Inhibit the proliferation and migration cyclin A2, CDK2, p-AKT, p-p44/42 MAPK, and p-p38 MAPK genes expression | Breast cancer tissues | MCF-7 | qRT-PCR | ‑­­(Chen et al., 2017b) |
| LncRNA BANCR | ↑ | - | Poor prognosis | ER/PR/Her2 (+/-) breast cancer tissues | MDA‑MB‑231  MDA‑MB‑468 MCF-7 HCC1569  BT549 | qRT-CR | (Jiang et al., 2018b) |
| HOXA-AS2 | ↑ | miR-520c-3p | Poor prognosis | Breast cancer tissues | MDA-MB-453  MCF-7 | qRT-PCR | (Fang et al., 2017) |
| LncRNA HAGLR  LncRNA STK4-AS1  LncRNA DLEU7-AS1  LncRNA LINC00957  LncRNA LINC01614  LncRNA ITPR1-AS1 | ↑ | - | Poor prognosis | Breast cancer sample data from TCGA & Breast cancer tissues | - | Microarray,  qRT-PCR | (Zhong et al., 2017) |
| LncRNA LINC02163 | ↑ | miR-511-3p | Poor prognosis | ER/PR (+/-) breast cancer tissues | MDA-MB-231 SKBR-3  MCF-7  BT-474 | qRT-PCR | (Qin et al., 2020) |
| LncRNA HULC | ↑ | miR-6754-5p | Increase Migration & invasion | Breast cancer tissues | MDA-MB-231  MCF-7  ZR-75-1  BT-20 | qRT-PCR | (Wang et al., 2019c) |
| LncRNA LINC01614 | ↑ | - | Regulate TGF-β, & CDH1 genes expression | Breast cancer sample data from TANRIC | - | Microarray,  qRT-PCR | (Wang et al., 2019g) |
| LncRNA HEIH | ↑ | miR-200b | Regulate Wnt/β-catenin pathway | Breast cancer tissues | MDA-MB-231 | qRT-PCR | (Zhao et al., 2019a) |
| LncRNA PTENP1 | ↑ | miR-19b | Inhibit migration & invasion by regulating  PTEN/PI3K/Akt pathway | Breast cancer tissues | MDA-MB-231  MCF10A  MCF-7 | qRT-PCR | (Shi et al., 2018) |
| LncRNA RPPH1 | ↑ | miR-122 | Upregulate ADAM10, PKM2, NOD2, & IGF1R gene expression | Breast cancer tissues | MDA-MB-231  MDA-MB-453  HCC-1937  MCF-7 | qRT-PCR | (Zhang and Tang, 2017) |
| LncRNA NORAD | ↑ | - | Promote proliferation, invasion, and migration by regulating  TGF-β/RUNX2 signaling  pathway | Breast cancer tissues &  BALB/c female mice | MDA-MB-231 | qRT-PCR | (Zhou et al., 2019b) |
| LncRNA LINC00461 | ↑ | miR‑144‑3p | Upregulate KPNA2 gene expression | Breast cancer sample data from the TCGA database | MDA-MB-157  MDA-MB-231  AU565  MCF-7 | qRT-PCR | (Zhang et al., 2020c) |
| LncRNA SNHG22 | ↑ | miR‑324‑3p | Promote proliferation, invasion, and migration by regulating SNHG22/miR-324-3p axis | TNBC tissues | MDA-MB-231  MDA-MB-468  MDA-MB-436  HCC-1937 | qRT-PCR | (Fang et al., 2020) |
| LncRNA BLACAT1 | ↑ | miR‑150‑5p | Regulate CCR2 gene expression | ER/PR (+/-) breast cancer tissues | MDA-MB-231  SKBR-3 | qRT-PCR | (Hu et al., 2019) |
| LncRNA linc-ZNF469-3 | ↑ | miR-574-5p | Increase metastasis by regulating ZEB1 gene expression | Breast cancer tissues | MDA-MB-157 MDA-MB-231 MDA-MB-361 MCF7  BT483  AU565  SKBR-3  BT549  HCC1599 HCC1806 HS578T | ISH,  qRT-PCR | (Wang et al., 2018d) |
| LncRNA RNAZFHX4-AS1 | ↑ | FAT4 | Regulate the Hippo signaling pathway via modulating FAT4 gene expression | Breast cancer sample data from GSE3344 &  GSE2691 &  male BALB/c nude mice | MDA-MB-231 | qRT-PCR | (Li et al., 2019b) |
| LncRNA PRLB | ↑ | miR-4766-5p | Increase cell  growth, metastasis, chemoresistance & regulate SIRT1 expression | Nude female mice | - | Microarray,  qRT-PCR | (Liang et al., 2018b) |
| LncRNA LINC01125 | ↑ | - | Ani-tumour effects by regulating PTEN/Akt/p53 signaling pathway | Female BALB/c mice | MDA-MB-231  MCF-10A  BT549 | Microarray  qRT-PCR | (Wan et al., 2019) |
| LncRNA LINC02582 | ↑ | USP7 | Radio-resistance | - | MDA-MB-231  MCF-7  BT474  BT549  SKBR3  T47D | ISH,  qRT-PCR | (Wang et al., 2019a) |
| LncRNA LRRC75A-AS1 | ↑ | miR-380–3p | Increase proliferation, invasion, and EMT through LRRC75A-AS1/miR-380–3p/BAALC axis | Breast cancer tissues | MDA-MB-468 MDA-MB-436  MDA-MB-231  HCC-1937 | FISH,  qRT-PCR | (Li et al., 2020b) |
| LncRNA LINC01614 | ↑ | - | Poor prognosis, regulate TGF-β and FAK signaling | Breast cancer sample data from TANRIC | MDA-MB-231  MDA-MB-453  MDA-MB-468  BT474  T-47D  ZR751  MCF7  HCC70  HS578T  BT549 | RNA-Seq  qRT-PCR | (Vishnubalaji et al., 2019) |
| LncRNA LINC01133 | ↑ | miR-199a | Cancer Stem cell-Like  Phenotypic by regulating FOXP2 & KLF4 gene expression | - | MDA-MB-231  MDA-MB-468 HCC1937  T47D  MCF7  ZR-75-1  BT-20  HCC1143  BT549  Hs578T | qRT-PCR | (Tu et al., 2019) |
| LncRNA Linc00339 | ↑ | miR-377-3p | Proliferation through regulating HOXC6 expression, | Mice | MDA‐MB‐231 MDA‐MB‐468 | qRT-PCR | (Wang et al., 2019d) |
| LncRNA LINC00472 | ↑ | - | Tumour progression  and disease recurrence by regulating NF-kβ activation and expression | BALB/c female nude mice | MDA-MB-231  MCF-7  T47D  Hs578T  SKBR3 | qRT-PCR | (Wang et al., 2019i) |
| LncRNA HOXA‐AS2 | ↑ | miR‐106a | Tumour progression by regulating SCN3A gene expression | Breast cancer sample data from TCGA | - | Microarray,  qRT-PCR | (Wu et al., 2019) |
| LncRNA BCHE | ↑ | - | Tumour progression by regulating ITGB1 gene expression | Breast cancer tissues | MDA-MB-231  MDA-MB-468  MCF-7 | qRT-PCR | (Yang et al., 2018a) |
| LncRNA ADPGK-AS1 | ↑ | miR-3196 | Increase proliferation, EMT process, and suppress apoptosis by miR-3196/OTX1 axis | Breast cancer tissues | MDA-MB-436  MDA-MB-453  MDA-MB-231  MCF-7 | qRT-PCR | (Yang et al., 2019a) |
| LncRNA sONE | ↑ | miR‐34a miR‐15  miR‐16 & let‐7a | Regulate p53/c‐Myc signaling pathway | ER/PR (+/-) breast cancer tissues | MDA‐MB‐231 MCF‐7 | qRT-PCR | (Youness et al., 2019) |
| LncRNA FEZF1-AS1 | ↑ | miR-30a | Regulate Nanog, Oct4, & SOX2 genes expression | ER/PR (+/-) breast cancer tissues &  BALB/c mice | MDA-MB-231  MDA-MB-468 MDA-MB-453  MCF-7 | qRT-PCR | (Zhang et al., 2018) |
| LncRNA RHPN1‑AS1 | ↑ | miR-4261 | Regulating the expression of  c-Myc, & p53 genes expression | Breast cancer tissues | MCF-7 | qRT-PCR,  FISH | (Zhu et al., 2019a) |
